# Membrane Separation Techniques for Plant Essential Oils: Theory, Performance Comparison, and Application—An Updated Review

**DOI:** 10.3390/foods15132283

**Published:** 2026-06-25

**Authors:** Yiheng Xiao, Yahan Fu, Yifan Bu, Letian Tang, Jinyang Wang, Haobo Zhang, Qiang Li, Changxia Sun

**Affiliations:** 1 Department of Chemistry, College of Science, Beijing Forestry University, Beijing 100083, China; 19004505777@163.com (Y.X.);; 2 College of Environmental Science and Engineering, Beijing Forestry University, Beijing 100083, China; byf1020@bjfu.edu.cn (Y.B.); 18949076317@163.com (H.Z.)

**Keywords:** plant essential oil, membrane separation technology, essential oil extraction, membrane purification

## Abstract

Plant essential oils are widely utilized as natural preservatives, flavoring agents, and nutritional supplements owing to their remarkable antibacterial, antioxidant, and aroma-enhancing properties. However, their low abundance in plant matrices, together with the compositional complexity and thermal sensitivity of volatile constituents, poses significant challenges for efficient extraction and purification. In recent years, membrane separation technology has emerged as a promising green strategy for the extraction, purification, and concentration of plant essential oils. Membrane-based processes, including microfiltration, ultrafiltration, nanofiltration, reverse osmosis, and pervaporation, enable selective separation under mild operating conditions based on differences in molecular size, polarity, and diffusivity. Compared with conventional thermal- and solvent-based methods, membrane processes offer lower energy consumption, reduced solvent usage, and superior retention of thermolabile bioactive compounds and natural aroma profiles. Moreover, recent advances in membrane materials and surface modification strategies have significantly improved membrane selectivity, permeability, and fouling resistance, thereby enhancing process stability and industrial applicability. This review systematically summarizes the theoretical principles, separation mechanisms, membrane classifications, and recent applications of membrane technologies in plant essential oil processing. Based on a comparative analysis of more than 120 published studies, the performance of different membrane processes is evaluated in terms of flux, selectivity, energy consumption, and product quality. Particular attention is given to current challenges, including the lack of standardized performance metrics and comprehensive techno-economic assessments. Recent advances in membrane materials and surface modification strategies, together with future research directions and industrial prospects, are also discussed. This review provides valuable guidance for membrane selection, process optimization, and sustainable industrial implementation in plant essential oil extraction and purification.

## 1. Introduction

**Figure 1:**
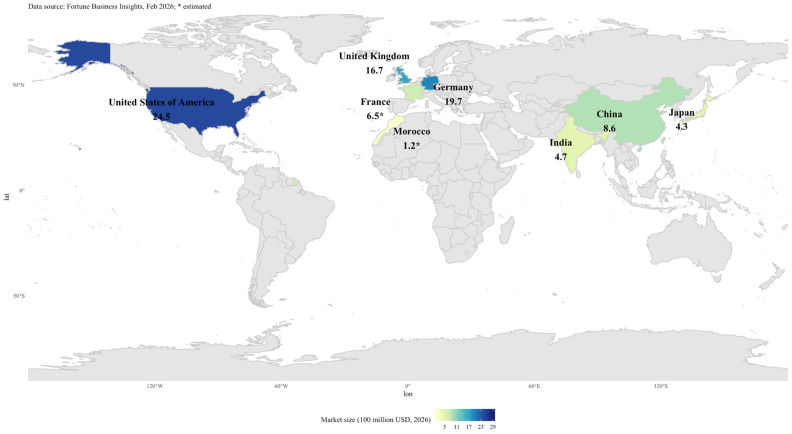
Estimated market size distribution of essential oils in major countries and regions around the world in 2026.

Essential oils, derived from various plant tissues such as flowers, leaves, stems, roots, fruits, and seeds, are volatile aromatic compounds with significant biological properties. These oils primarily consist of bioactive compounds like terpenes, alcohols, phenols, aldehydes, and esters, which possess a wide array of beneficial physiological functions, including antimicrobial, antioxidant, anti-inflammatory, sedative, insect-repellent, and preservative effects [1,2]. Owing to their natural origin, mild properties, and appealing aromatic profiles, plant essential oils have found widespread application in diverse industries, such as cosmetics, food flavoring, pharmaceuticals, healthcare products, aromatherapy, and agricultural pest control [3,4,5]. Recent market analyses indicate that the global essential oils market is expected to reach approximately USD 15.01 billion by 2026 and further increase to USD 34.8 billion by 2034, representing a compound annual growth rate of approximately 11.08% over the forecast period (as shown in Figure 1). This rapid growth highlights the increasing demand for essential oils across a wide range of sectors, including pharmaceuticals, food, cosmetics, and aromatherapy. With increasing consumer preference for natural and environmentally friendly products, the global market for plant essential oils is growing, presenting significant development potential and economic value [6].

However, the industrial extraction and purification of essential oils present several challenges (as shown in Figure S1), particularly when it comes to efficiently isolating bioactive components from complex crude extracts. These crude oils are typically contaminated with various impurities, including plant debris, macromolecular proteins, polysaccharides, residual extraction solvents, and excessive moisture. Traditional purification methods, such as distillation, solvent extraction, filtration, and chromatography, often face limitations. Steam distillation is one of the most widely used methods for extracting essential oils from plants, but it can lead to the loss of heat-sensitive compounds. Substantial evidence supports its dominant industrial role. Similarly, Machado et al. (2022) identified steam distillation as the most commonly used extraction method due to its simplicity and low investment cost, and an analysis of 490 patent documents confirmed its continued industrial relevance and expanding applications [7]. Solvent extraction, as applied by Olmedo et al. (2014) for isolating essential oils from Origanum vulgare (oregano), may result in residual solvent contamination in the final product and generally requires high energy input [8]. H. Rassem et al. (2016) also employed solvent extraction and noted its relatively mild operating conditions and lower cost compared with heat-intensive methods such as steam distillation [9]. In addition, chromatographic techniques such as high-performance liquid chromatography (HPLC) are used for essential oil separation and analysis but are often costly and time-consuming. For example, C. Turek & F. Stintzing, 2011 established an HPLC method with diode array detection and mass spectrometry to analyze characteristic compounds in eucalyptus essential oil, demonstrating fingerprint analysis at 220 nm [10]. C. Cagliero et al. (2021) reviewed the latest developments in gas chromatography (GC) for essential oils, reaffirming that the progress of GC remains closely intertwined with the challenges of essential oil research, from both industrial and scientific standpoints [11]. Overall, these conventional techniques are typically energy-intensive, time-consuming, and may involve solvent loss or thermal degradation of sensitive compounds. In addition, they often fail to fully meet modern industrial requirements for high purity, efficiency, and environmental sustainability [12,13].

In this context, membrane separation technology has emerged as a promising and eco-friendly solution for the refining of plant essential oils [14]. The technology relies on the selective permeability of membranes, with pressure and concentration differences serving as the driving forces for the separation of essential oil components from impurities. Membrane separation relies on two distinct mechanisms. First, the membrane’s pore size defines a physical cutoff—only particles smaller than the pores can pass through, while larger ones are retained [15]. Surface properties (hydrophilicity, hydrophobicity, and charge) modulate selective permeation: hydrophilic surfaces facilitate water passage and resist fouling, hydrophobic ones tend to adsorb nonpolar solutes, and charged surfaces repel or attract ionic species via electrostatic forces. Together, pore sieving sets the size threshold, while surface chemistry governs affinity-based selectivity [16]. This process consists of two synergistic aspects. On one hand, the membrane pore size determines a physical cutoff, defining which particles are retained or allowed to permeate based on size [17]. On the other hand, surface properties—hydrophilicity, hydrophobicity, and charge—govern selective permeation by mediating molecular-level interactions with different components [18]. The membrane-based extraction of plant essential oils operates under mild, non-thermal conditions, enabling high retention of thermosensitive bioactive and volatile aroma compounds with minimal energy input. This selective permeation strategy inherently aligns with green chemistry and the sustainable processing of natural plant resources [19,20].

**Figure 2:**
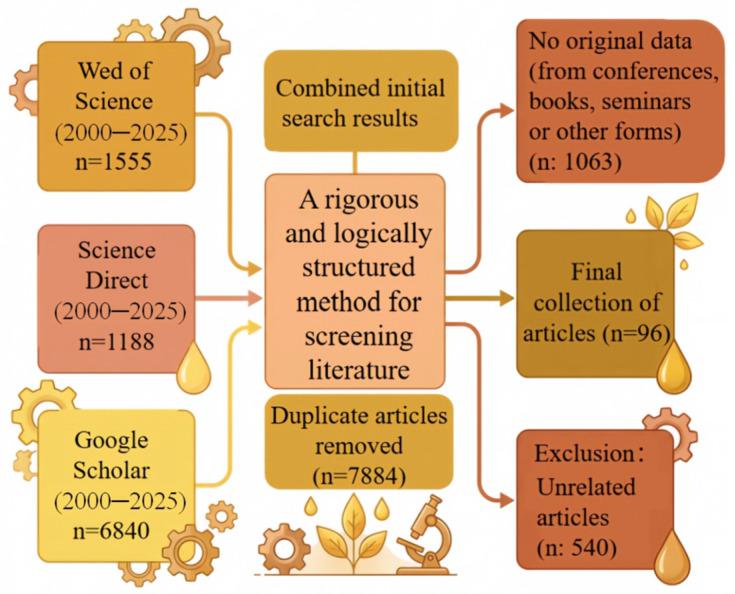
Literature related to the extraction of plant essential oils using membrane filtration technology.

While membrane separation offers clear benefits, previous reviews have typically addressed only isolated membrane types or specific mechanisms and are now outpaced by recent advances; this updated review is the first to provide a systematic, critical synthesis of membrane materials, transport mechanisms, separation performance, and industrial feasibility specifically for essential oil refining, thereby presenting a timely and novel perspective beyond earlier, fragmented assessments. As shown in Figure 2, a total of 9583 records published between 2000 and 2025 were initially identified from Web of Science (*n* = 1555), ScienceDirect (*n* = 1188), and Google Scholar (*n* = 6840). After conducting a systematic literature search was conducted in major scientific databases using the Boolean search query (“plant essential oil”) AND (membrane OR “filtration membrane”). After removing duplicate records, non-original publications such as conference papers, books, and seminar proceedings were excluded. The remaining studies were further screened for relevance to membrane-based extraction and purification of plant essential oils. Following full-text assessment, a total of 96 eligible studies were finally included in this review. The selection of these 96 articles followed the following principles: Only original studies that contain directly extractable quantitative data were included. As shown in Figure S2, using CiteSpace, we analyzed the temporal evolution of research themes and project emphases in the field of plant essential oil extraction using membrane technology. The results of our literature analysis revealed three distinct developmental stages in membrane separation technologies for plant essential oil processing. From 1950 to 2000, research primarily focused on pervaporation and vapor permeation, reflecting the early emphasis on the separation of volatile compounds using dense membrane systems. Between 2000 and 2020, the research scope expanded considerably, with pervaporation, vapor permeation, and ultrafiltration emerging as the dominant membrane technologies. Since 2020, research attention has progressively shifted toward ultrafiltration and nanofiltration, highlighting the growing interest in low-pressure, high-selectivity membrane processes for the efficient recovery and purification of plant essential oils. These findings, summarized from the collected literature, demonstrate the evolving research priorities and technological trends in membrane-based essential oil processing. This review provides an updated overview of membrane separation technology in plant essential oil extraction. It first discusses the fundamental principles and mass transfer mechanisms of membrane processes used for oil purification. Subsequently, the review categorizes commonly used membranes, such as microfiltration (MF), ultrafiltration (UF), nanofiltration (NF), reverse osmosis (RO), and pervaporation (PV), outlining their structural characteristics, operational performance, and applications in essential oil processing. In addition, a comparison of the separation efficiency, purification effectiveness, operational stability, and economic feasibility of different membrane technologies is provided, with an emphasis on recent advances in the field. It also highlights the limitations and challenges associated with applying membrane separation technology at an industrial scale, and suggests potential optimization strategies. This work aims to offer valuable insights to researchers and industry professionals, facilitating the selection of suitable membrane technologies and promoting the sustainable development of the essential oils industry.

## 2. Fundamental Mechanisms of Membrane Separation in Plant Essential Oil Processing

**Figure 3:**
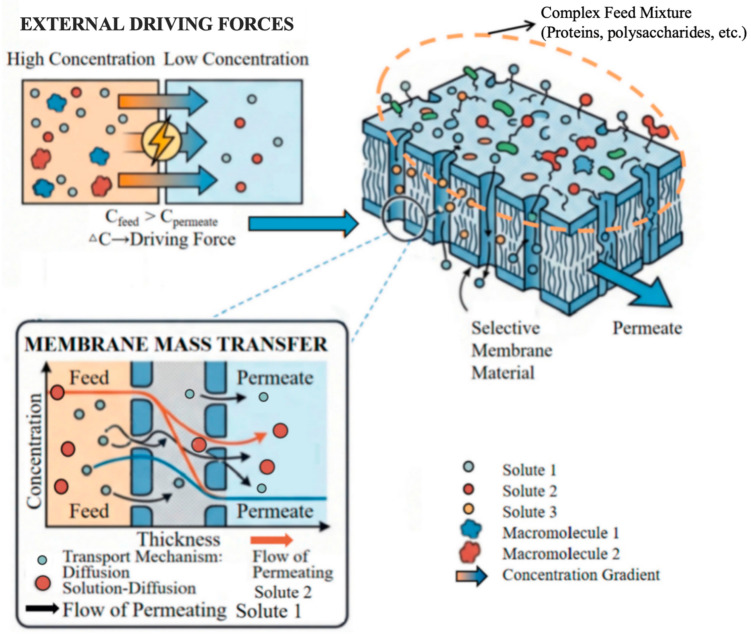
Separate Plant essential oils by using selective permeable membrane materials and external driving forces.

Membrane separation technology encompasses a set of processes in which external driving forces—such as pressure differentials, concentration gradients, electrochemical potentials, or temperature gradients—are applied to achieve the separation, purification, or concentration of distinct components within a mixed system using selectively permeable membrane materials [21]. In these processes, the membrane serves as a selective barrier between the feed and permeate phases. As shown in Figure 3, driven by one or more forces, permeating components are transported from the high-potential feed phase to the low-potential permeate phase. The concentration gradient, a key mechanism, results from the synergy between the external driving force and the selective membrane material, where the former supplies the necessary energy for mass transfer, and the latter determines the separation efficiency and selectivity [22]. The application of different driving forces endows membrane separation processes with distinct mechanistic features and application scenarios. As studied by Alkhudhiri et al. (2012), MF and UF are pressure-driven processes that achieve separation primarily through size exclusion [23]. RO and NF are also pressure-driven, while they use dense membranes where transport follows the solution-diffusion mechanism, and the required applied hydraulic pressure must exceed the osmotic pressure of the feed to drive permeation [24]. Furthermore, technologies like membrane distillation (MD) with high theoretical rejection rates, driven by vapor pressure differences, and holds potential applications across various industries, have shown considerable promise for the recovery of volatile essential oils [25]. This section aims to classify membrane separation processes based on physical and chemical principles and membrane structures, providing a comprehensive discussion on the theoretical foundations, recent progress, and application characteristics of membrane separation in essential oil extraction.

### 2.1. Classification of Physical Mechanisms

**Figure 4:**
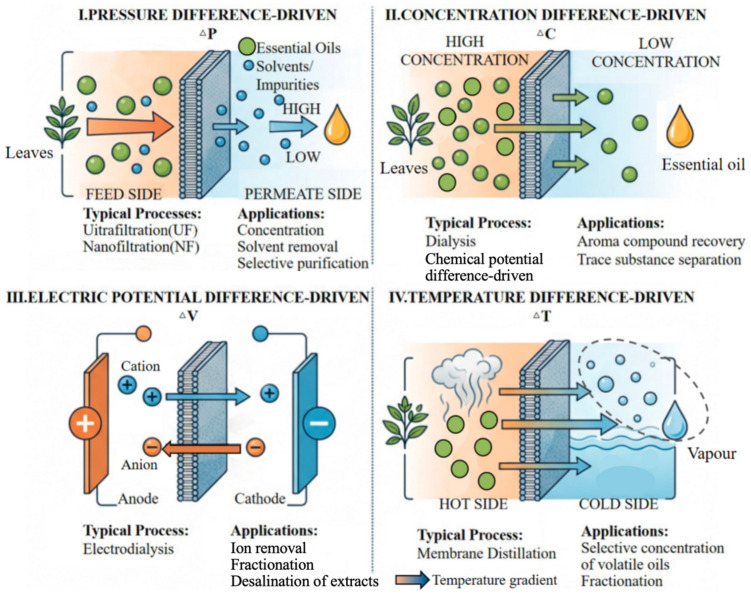
The physical mechanism of membrane separation in the extraction process of plant essential oils.

In the extraction of plant essential oils, the physical mechanisms of membrane separation are defined by external forces that govern solute permeation or retention. As shown in Figure 4, these forces are mainly classified into four categories: pressure difference, concentration difference, potential difference, and temperature difference. Below, the theoretical basis, representative membrane processes, and their applications in essential oil extraction are discussed [26].

#### 2.1.1. Pressure-Driven Separation: The Sieving Mechanism

This process is primarily governed by the transmembrane pressure difference (TMP). When the pressure on the feed side exceeds that on the permeate side, components smaller than the membrane pores permeate, while larger species are retained. Under this pressure gradient, both the solvent and permeable solutes migrate toward the permeate side, whereas impermeable substances are excluded through pore-size-dependent sieving. As demonstrated by Bolto et al. (2020), the incorporation of MF or UF as a pretreatment step in essential oil extraction can significantly enhance the efficiency of subsequent membrane or extraction processes while preserving the integrity of aromatic compounds [27]. This mechanism underpins MF and UF and makes them particularly effective for removing suspended solids, plant debris, oil–water emulsions, and waxy precipitates, thereby producing a clarified essential oil solution or concentrate.

#### 2.1.2. Concentration Gradient-Driven Mechanism: The Solution–Diffusion Process

This mechanism exploits the concentration gradient—or chemical potential difference—of solutes or solvents across the membrane. Permeating species first dissolve into the membrane matrix, diffuse through it, and subsequently desorb on the downstream side. This behavior is predominantly observed in NF, RO, and MD. Theoretically, such processes are described by the solution–diffusion model or the coupled diffusion–convection model. In plant essential oil extraction, membranes operating under this mechanism can be employed to concentrate aromatic small molecules, remove soluble impurities (e.g., sugars, amino acids, polar phenols), or efficiently dehydrate and concentrate aqueous essential oil solutions. Olkiewicz et al. (2023) demonstrated that membrane-based processes can promote the concentration and separation of essential oils during extraction, thereby improving recovery efficiency and product purity [28]. Thus, the concentration-gradient-driven membrane process offers molecular-level selectivity and mild operational conditions, positioning it as a critical technological link between crude extraction and refined processing.

#### 2.1.3. Electric Potential Gradient-Driven Mechanism: Electro-Membrane Separation

This mechanism relies on an applied electric field across ion-selective or charged membranes, where charged species migrate according to their valence and mobility under the potential gradient. Unlike pressure-driven processes that rely on size exclusion, electro-membrane separation—exemplified by electrodialysis (ED) and membrane electrophoresis—exploits differences in ionic charge and electrochemical potential [29,30]. When a direct current field is established, cations move toward the cathode and anions toward the anode, allowing selective enrichment or depletion of charged compounds from feed streams. In the context of plant essential oil extraction, this mechanism finds particular utility in removing ionic impurities—such as organic acids, salts, and certain polar phenolic derivatives—from crude essential oil emulsions or hydrosols, especially when a high-purity, non-polar oil phase is desired [31]. As shown by Taylor (1996) in the study of electrically enhanced phase separation of water-in-oil emulsions, high-intensity AC electric fields can destabilize emulsions effectively, offering a non-thermal, solvent-free approach that is potentially applicable to the recovery of polar or charged species from essential oil emulsions [32]. Moreover, electro-membrane processes operate at ambient temperature and low pressure, offering a mild alternative for heat-sensitive aromatic molecules. Thus, the potential-gradient-driven approach complements pressure- and concentration-driven methods by tackling charged contaminants that are otherwise difficult to remove by sieving or passive diffusion alone [33].

#### 2.1.4. Temperature Gradient-Driven Mechanism: Membrane Distillation

MD utilizes temperature gradients or vapor pressure differences to drive the thermal separation process; only vapour molecules pass through the microporous hydrophobic membrane [34]. Wang et al. (2020) investigated low-temperature membrane distillation for volatile or thermally sensitive essential oil components and demonstrated its ability to minimize thermal degradation [35]. Unlike traditional distillation, membrane distillation couples membrane technology with temperature gradients, representing an innovative trend in essential oil purification. This mechanism is particularly effective in dehydration concentration, volatile component enrichment, and low-heat regeneration stages, thereby improving product purity while minimizing heat-induced damage.

### 2.2. Classification of Chemical Mechanisms

In addition to physical driving forces, chemical interactions between membrane materials and target solutes are crucial for determining the selectivity and efficiency of membrane separation. This section elaborates on the chemical mechanisms that govern essential oil extraction processes, particularly the interactions between essential oil components and membrane surfaces, including the hydrophilic or hydrophobic properties of membranes, and the influence of membrane modifications [36,37].

#### 2.2.1. Hydrogen Bonding and Chemical Bonding Between Essential Oil Components and Membrane Surfaces

Functional groups on the membrane surface or within its pores can interact with polar groups, heteroatoms, or aromatic ring structures in essential oils via hydrogen bonding, van der Waals forces, and π-π interactions [19]. Bolto et al. (2020) investigated these chemical bonding mechanisms and found that, while such interactions can enhance the membrane’s enrichment of target aromatic molecules, they may also reduce permeate flux or cause fouling due to membrane-solute interactions [27]. I. Sadeghi & A. Asatekin, 2019 directly demonstrated π-π interactions between aromatic molecules and phenol-functionalized pore walls, showing the aromatic molecule permeated 7.1 times faster than its nonaromatic analogue in competitive diffusion [38]. Therefore, membrane material design must strike a balance between solute affinity and permeation efficiency.

#### 2.2.2. Hydrophilicity/Hydrophobicity Interactions

Essential oils are typically volatile and hydrophobic. The hydrophilic or hydrophobic nature of membranes significantly influences separation behavior. Hydrophobic membranes, for instance, preferentially facilitate the permeation of lipophilic aromatic oil components, enriching the oil phase. Chang Gao et al. (2025) designed a PVDF/POMAA membrane using surface separation and response surface methodology (RSM), achieving an oil retention rate of 92.4% whilst maintaining a high water flux; this validated the partitioning principle without undue simplification [39]. In contrast, hydrophilic membranes are better suited for oil removal or clarification in aqueous phases.

#### 2.2.3. Functional Membrane Modification for Catalysis, Carrier Function, or Selectivity Enhancement

Membranes can be modified to incorporate catalytic enzymes, affinity ligands, or functional nanoparticles, enabling integrated extraction-conversion-separation processes. For example, Rangaswamy (2021) discussed membrane reactors loaded with enzymes or catalysts to enhance the extraction and enrichment of target components [37]. Such innovations are gaining increasing attention for their potential in natural product separation.

### 2.3. Synergistic Mechanisms of Physics and Chemistry

In essential oil extraction, the effectiveness of membrane separation arises from the synergy between physical and chemical mechanisms. Membrane flux, selectivity, and anti-fouling performance are influenced by external driving forces (e.g., pressure differentials and concentration gradients) and membrane-solute interactions (e.g., hydrogen bonding, hydrophobic interactions). Membrane separation systems, therefore, integrate physical mass transfer with chemical affinities, ultimately determining effective selectivity. For instance, hydrophobic essential oil molecules form adsorption layers on hydrophobic membrane surfaces, enhancing selective permeability, while polar impurities are more easily repelled by hydrophilic membranes [19,27].

**Figure 5:**
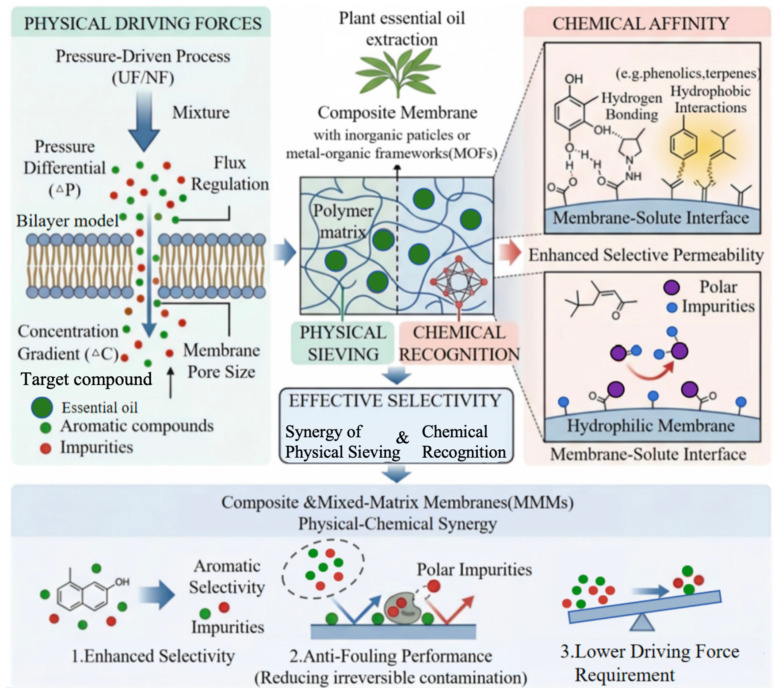
The Physical and Chemical Combined Sieving Mechanism of Membrane Separation in the Extraction Process of Plant Essential Oils.

Composite and mixed-matrix membranes (MMMs), incorporating inorganic particles or metal–organic frameworks (MOFs), offer a promising platform for optimizing both physical and chemical properties, enhancing membrane performance and sustainability in essential oil extraction. Bhoga Arundhathi et al., (2024) confirm that “NPs exert a profound influence on membrane performance, enhancing both permeability and selectivity simultaneously.” [40]. Jaid et al. (2024) demonstrated that mixed matrix membranes (MMMs) incorporating metal–organic frameworks (MOFs), covalent organic frameworks (COFs), and hydrogen-bonded organic frameworks (HOFs) provide a promising platform for developing stable and highly effective gas and liquid separation materials, with significant potential for applications in water treatment and gas separation processes [41]. As shown in Figure 5, their potential applications in essential oil extraction are primarily threefold: (1) enhancing the membrane’s selective permeability, (2) reducing irreversible fouling caused by adsorption–desorption cycles, and (3) maintaining high separation efficiency under lower driving forces. Recent reviews have indicated that composite membrane structures can effectively achieve the dual functions of physical sieving and chemical recognition [42,43].

### 2.4. Synergistic Effects in Membrane Separation

In conclusion, the synergistic effects of physical and chemical mechanisms, coupled with membrane structure design, form the foundation of membrane separation technology, enabling the achievement of high throughput, selectivity, and low energy consumption in plant essential oil extraction [42,43]. Due to these reasons, future research might focus on quantitatively analyzing the contributions of physical and chemical forces through material design and process optimization, thereby refining membrane structures and operational conditions. These efforts will not only advance scientific understanding but also provide innovative solutions for the green, high-purity extraction of food-grade essential oils.

## 3. Performance Evaluation of Membrane Separation Technologies

### 3.1. Comparison of Membrane Separations with Traditional Technologies

Conventional extraction of essential oils relies on hydrodistillation, steam distillation, cold pressing, and solvent extraction. These methods typically deliver low yields (0.5–5 wt.%), require high energy input (2–10 kWh per kg of oil), and expose heat-sensitive compounds to thermal degradation [44,45]. Membrane-based processes summarised in Table 1 operate at substantially lower temperatures and achieve higher recovery rates with better preservation of native bioactive properties [46].

Recovery efficiency. Hydrodistillation generally recovers 60–80% of volatile aroma components after condensation and phase separation [47]. In contrast, pervaporation (PV) using a ZIF-8/PDMS composite membrane recovered ~86% of total aromatics from lemon oil wastewater, with individual component recoveries of 78–92% (No. 1) [48], and PV with a PERVAP 1060 PDMS membrane achieved 90–95% recovery of apple aroma at 60 °C (No. 2) [49]. Organic solvent nanofiltration (OSN) with Duramem™ membranes retained >95% of total antioxidant capacity from rosemary extract (No. 4) [50].

Operating temperature and product quality. Traditional distillation operates near 100 °C, which can cause losses of 30–50% of oxygenated monoterpenes and reduce antioxidant activity [45]. Membrane processes in Table 1 are performed between 20 and 60 °C. For example, orange juice aroma was recovered by PV at 42 °C (No. 8) [51], and rosemary extract was concentrated by OSN at 25 °C (No. 4) [50]. This low-temperature operation preserves bioactivity: the ZIF-8/PDMS membrane retained >90% of antioxidant components (limonene, geraniol) from lemon oil (No. 1) [48], and the integrated UF–OD–PV process for kiwifruit juice resulted in only a ~12% decrease in total antioxidant activity (No. 6) [52].

Fouling and stability. Membrane fouling is manageable under appropriate conditions. The PDMS hollow fiber module for orange juice aroma maintained stable flux with minimal concentration polarization (No. 8) [51]. For supercritical CO_2_ separation of nutmeg essential oil, the cellulose acetate RO membrane showed reversible fouling and full flux recovery after depressurisation (No. 10) [53]. Membrane service life spans from >0.5 years to 4 years (Table 1).

Cost. PV membrane fabrication costs fall between 20 and 50 USD m^−2^ (Nos. 1, 2, 7, 8) [48,49,51,54]; NF and OSN membranes range from 30 to 150 USD m^−2^ (No. 4) [50]. Although traditional distillation stills may have lower capital expenditure, the combination of higher product quality, reduced energy consumption, and modular scalability renders membrane processes economically competitive for essential oil and aroma recovery.

**Table 1:** Key performance indicators of representative high-efficiency membrane processes for essential oil and aroma recovery.

No.	Reference (Authors, Year)	Membrane Type	Extract/Aroma Source	Principle	Total Flux (g·m^−2^·h^−1^ or L·m^−2^·h^−1^)	Feed Temp. (°C)	Fabrication Cost (USD·m^−2^)	Service Life	Antifouling/Stability	Component Recovery/Enrichment Performance	Total Recovery/Retention	Ref.
1	Dongyi Yang et al. (2023)	ZIF-8/PDMS composite membrane	Lemon oil wastewater (Citrus EO)	PV	560–720 g·m^−2^·h^−1^	40	20–50	>0.5 yr	Flux recovery ≈98% after cleaning; enhanced hydrophobicity	Limonene 92%, β-pinene 85%, γ-terpinene 88%, Geraniol 78%	≈86%	[48]
2	Hornyák et al. (2014)	PERVAP 1060 PDMS flat-sheet membrane	Apple aroma model solution	PV	100–500 g·m^−2^·h^−1^	20–60	20–50	>0.5 yr	Membrane resistance dominant; minimal liquid phase resistance; stable flux	β: ethanol 15.9–96.1, i-butanol 4.1–64.7, ethyl acetate 1.2–7.0	90–95% (highest at 60 °C)	[49]
3	Abdolreza Aroujalian et al.	PDMS-PVDF-PP composite membrane	Fresh orange juice (Citrus EO)	PV	100–500 g·m^−2^·h^−1^	/	200–500	2–4 yr	Hydrophobic; certain antifouling; regular cleaning required	β: Ethyl acetate ~18, Ethyl butyrate 4–10, Limonene 2–6	/	[55]
4	Peshev et al. (2011)	Duramem™ OSN membranes (200/300/500 Da)	Rosemary ethanol extract	OSN	15.4–41.1 L·m^−2^·h^−1^	25 ± 1	80–150	>0.5 yr	Flux decay ≈15% within 2 diavolumes	RA ≈99.2%, CA ≈95%	>95% (total antioxidant retention)	[50]
5	Castro-Muñoz (2024) (Review)	NF270, NF90, Desal DK/DL, Duramem, etc. commercial NF membranes	Polyphenols/carotenoids (wine pomace, olive wastewater, citrus, berries, etc.)	NF	5–50 L·m^−2^·h^−1^	20–45	30–80	>0.5 yr	Fouling/CP resistance 20–80%; recoverable by cleaning	Rejection: phenolics/anthocyanins 88–100%; carotenoid concentration 90–99%	80–98% (integrated MF/UF + NF → ≈100%)	[56]
6	Cassano et al. (2006)	UF (PVDF) + OD (PP) + PV (PDMS) integrated process	Kiwifruit juice aroma	UF → OD → PV integrated	UF: 15.6 → 7 L·m^−2^·h^−1^; OD: 1000 → 470 g·m^−2^·h^−1^; PV: 0.72–1.8 g·m^−2^·h^−1^	UF/OD 25; PV 20–40	20–50	/	UF slight polarization recoverable > 95%; OD stable	β (esters) ≈ 100, β (alcohols) 10–40	Total aroma retention 90–95%	[52]
7	Sampranpiboon et al. (2000)	PDMS composite membranes	Simulated fruit EO (ethyl butyrate, ethyl hexanoate)	PV	ETB: 10–19 g·m^−2^·h^−1^; ETH: 13–24 g·m^−2^·h^−1^	30	20–50	/	PDMS slightly better; PDMS concentration polarization more obvious	β: PDMS 118–281, PDMS 77–234; ETH recovery up to 85–90%	≈85–90% (ETH)	[54]
8	Shepherd et al. (2002)	PDMS hollow fiber membrane (WSLO and transverse flow modules)	Orange juice aroma (Citrus EO)	PV	Aroma: 7–8 g·m^−2^·h^−1^; Water + ethanol: 20–30 g·m^−2^·h^−1^	42	20–50	>0.5 yr	WSLO module reduces concentration polarization; stable flux	β: Ethanol 3.8, Aroma ≈ 8; Ethyl butyrate conc. ~50 ppm	≈80%	[51]
9	Pereira et al. (2005)	Pervap 1060/1070, EPDM, EVA flat-sheet/HF membranes	Pineapple juice aroma model solution	PV	Water flux: 12.7–94.4 g·m^−2^·h^−1^ (membrane dependent)	25	20–60	/	EPDM/EVA low water flux, low polarization; membrane resistance dominant	β: EPDM-HF EA 125.3, EB 516, EH 1161; Pervap 1070 EA 124.4, EB 410	66–90% (engineering simulation)	[57]
10	Spricigo et al. (2001)	Cellulose acetate RO membrane (CF, Osmonics)	Nutmeg EO/dense CO_2_	RO (dense CO_2_)	CO_2_ permeability 32.1 kg·h^−1^·m^−2^·MPa^−1^ (flux linear with ΔP)	23–50	20–60	≥180 h	Reversible fouling/CP; pure CO_2_ flux recoverable	Average retention index 96.4% (±1.4%); composition unchanged	/	[53]

### 3.2. Analysis of Key Factors in Membrane Separation Technology

Through a comparative analysis of nearly 20 academic publications (summarized in Table S1), the performance of membrane-based processes for plant essential oil separation was systematically analyzed with respect to chemical composition, membrane characteristics (effective area, flux, and lifespan), and economic considerations. The main findings are summarized as follows.

From the perspective of raw material classification, the selection of membrane processes and materials is highly dependent on the chemical composition and polarity of essential oils. For citrus essential oils (e.g., lemon, orange, bergamot), NF is generally identified as the most suitable technique. Recommended membrane materials include polyamide (PA), polyethersulfone (PES), and polydimethylsiloxane (PDMS), with the latter being particularly suitable for organic solvent systems [58,59]. The primary application is deterpenation—the removal of large quantities of terpenes (e.g., limonene)—and the concurrent enrichment of oxygenated terpenes (e.g., linalool, nerolidol), which possess superior flavor profiles [60].

For spice essential oils (e.g., black pepper, cinnamon, clove, ginger). Organic solvent nanofiltration (OSN) membrane technology is most effective for spice essential oil extraction. Recommended membrane materials include polyimide (PI), cross-linked PA, PDMS composite membranes, and PES [59]. This technology is applicable to essential oil processing in high-polarity or non-polarity solvent systems, specifically for terpene removal, decolorization, and fractional concentration. It is particularly well-suited for extracting high-purity pungent active compounds such as piperine, cinnamaldehyde, and eugenol [58,61].

For herb essential oils (e.g., rosemary, mint, basil, thyme). Chaudhari et al. (2025) identified that OSN and NF are the most suitable membrane technologies for herb-derived essential oils, and recommended PA, PES, PDMS-based composite membranes, and PI as effective membrane materials [62]. These processes are primarily used for the enrichment and purification of bioactive constituents (e.g., rosmarinic acid, menthol, and thymol), and exhibit good compatibility with both aqueous and organic phase systems [63].

For floral essential oils (e.g., rose, jasmine, chamomile, lavender). Silvestre et al. reviewed pervaporation for separating essential oil components and recommended PDMS and its modified composites, PI, and PE as effective membrane materials [64]. These techniques are particularly effective for the selective enrichment of volatile aroma compounds (e.g., phenylethyl alcohol and lactones) under mild or solvent-free conditions [65,66,67,68].

Beyond separation performance, membrane processing also plays a critical role in preserving the bioactivity of essential oils. Due to the absence of high-temperature treatment, membrane-based processes better retain antioxidant compounds compared with conventional techniques. Other compounds, including camphor, citral, citronellal, rosmarinic acid, and linalyl acetate, further enhance the functional properties of essential oils. Although non-volatile polyphenols and flavonoids are not typical essential oil components, their partial co-extraction in certain systems may significantly improve antioxidant capacity [69,70].

From a process engineering perspective, membrane flux and fouling behavior are critical determinants of system performance. The concept of critical flux is particularly important: below this threshold, fouling is minimal, whereas above it, fouling increases sharply. Membrane type strongly influences flux characteristics. MF membranes, with pore sizes of 0.1–10 μm, exhibit the highest flux and are primarily used for clarification and removal of suspended solids. UF membranes (0.01–0.1 μm) enable the separation of macromolecules such as proteins and colloids [71,72,73]. NF membranes (0.001–0.01 μm) provide selective retention of small organic molecules and certain ions, while reverse RO membranes (<0.001 μm) offer the highest rejection but the lowest flux, limiting their applicability in essential oil processing where high throughput is required [65].

Economic considerations further influence technology selection. UF membranes generally offer a favorable balance between cost and lifespan (typically 3–7 years), resulting in relatively low long-term costs [72]. Although MF membranes are inexpensive, they are often used in combination with UF, reducing their standalone economic advantage [71]. NF and RO membranes are more costly without a proportionate increase in service life [61,74]. Surface modification strategies—including hydrophilic coating, nanomaterial incorporation (e.g., TiO_2_, SiO_2_), and charge regulation—have been widely explored to improve anti-fouling performance and extend membrane lifespan [66].

### 3.3. The Limitations of Membrane Separation Technology

#### 3.3.1. Despite Significant Advances and the Ongoing Shift from Laboratory Studies to Industrial Implementation, Membrane Separation Technology Continues to Face Critical Technical, Engineering, and Economic Constraints

Membrane fouling mechanisms and their effects on long-term flux stability and separation performance when processing complex essential oil systems have been studied [75]. The complex matrices of plant extracts—proteins, polysaccharides, tannins, pigments, and waxes—readily accumulate on membrane surfaces and within pores, causing flux decline and loss of separation efficiency [76]. For example, during ultrafiltration of kiwifruit juice, the permeate flux decreased from 15.6 to 7 L m^−2^ h^−1^ due to progressive fouling [52], illustrating the rapid performance decay that can occur even with clarified feeds. Routine chemical cleaning, while widespread, rarely restores the original membrane performance fully; each cleaning cycle introduces microscopic damage to the polymer matrix, which accumulates over months of continuous operation. This progressive and largely irreversible deterioration is seldom captured in short-term laboratory trials that typically report performance over only a few cleaning cycles. Concentration polarization phenomena, current mitigation strategies, and their associated limitations. Concentration polarization further exacerbates the problem by forming a gel-like boundary layer that increases mass transfer resistance [77]. The phenomenon is material-dependent: Sampranpiboon et al. observed that PDMS membranes exhibited more severe concentration polarization effects compared with PDMS membranes. During pervaporation of ethyl butyrate and ethyl hexanoate [54]. Increasing cross-flow velocity can reduce polarization, but the associated rise in pumping energy and the risk of shearing emulsified oil droplets impose practical limits on this strategy [78]. In addition, the trade-off between hydrophilicity, chemical resistance, and mechanical strength continues to restrict membrane material selection, especially for feeds with high viscosity or solids content [79,80]. The problem is particularly pronounced for essential oil systems containing natural waxes and resins: while the model solutions frequently used in academic studies may show minimal fouling (e.g., the stable flux reported for PDMS hollow fiber modules with orange juice aroma [51]), industrial feed streams with higher solids loading often exhibit markedly different fouling behavior that is not predicted by laboratory data [67,68].

**Figure 6:**
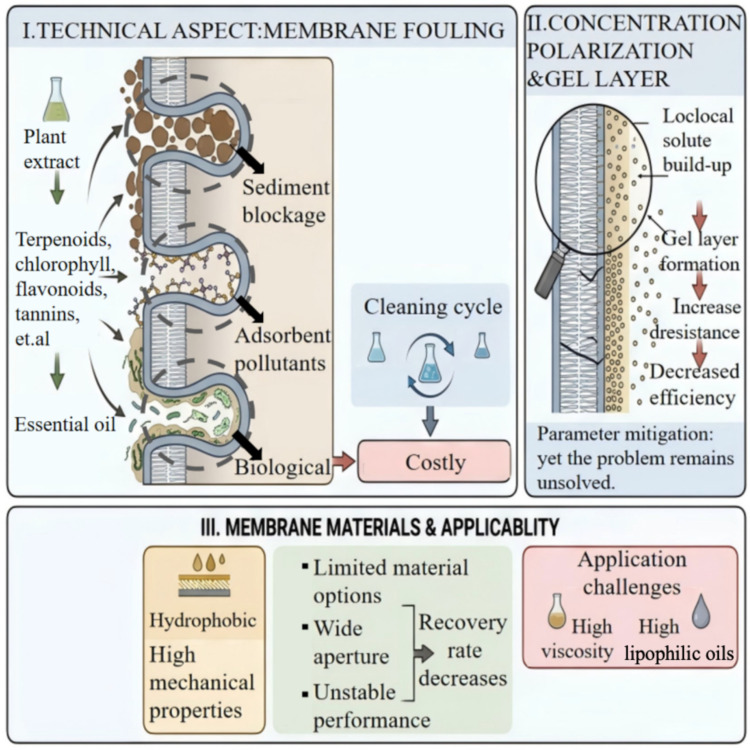
The Current Drawbacks of Extracting Plant Essential Oils Using Membrane Separation Technology.

As shown in Figure 6,at the engineering and operational level, membrane processes demand rigorous feed pretreatment and tight process control. Inadequate pretreatment—such as insufficient centrifugation or microfiltration—accelerates fouling and shortens membrane life [81]. Even minor fluctuations in pressure, temperature, or flow rate can shift membrane performance; for instance, in the pervaporation of apple aroma, the enrichment factor for ethanol varied from 15.9 to 96.1 depending on operating conditions. [49], demonstrating the sensitivity that necessitates precise and often costly control systems [82,83]. Furthermore, the scale-up from laboratory to industrial operation is non-trivial. Laboratory studies typically employ membrane areas of a few square centimeters to a few hundred square centimeters (as illustrated in Table 1), whereas industrial modules must accommodate several square meters to tens of square meters. The case of beer aroma recovery is illustrative: pervaporation tests were conducted using a 28 cm^2^ lab-scale membrane, whereas the conceptual industrial design required a membrane area of 13 m^2^ [84]. At such scales, flow maldistribution, uneven transmembrane pressure, and local flux variations become unavoidable, leading to accelerated fouling in stagnant zones and a total throughput substantially below the area-proportional projection from bench-scale data. Moreover, membrane longevity in real process environments is often shorter than the 0.5–5 year estimates obtained under controlled conditions; for example, PDMS/PTFE membranes used for peppermint aromatic water separation maintained excellent performance for the first 6 h, after which gradual wetting caused the separation factor reached a maximum of 668 under optimized conditions [85], underscoring the long-term stability challenges that are magnified in continuous industrial operation.

From an economic perspective, high initial capital investment continues to hinder adoption by small and medium-sized enterprises. Long-term industrial operation, with particular attention to the limited availability of continuous-operation and multi-cycle performance data, as well as the uncertainties associated with membrane lifetime, cleaning requirements, and operating costs. For example, the PDMS hollow fiber modules used for orange juice aroma recovery demonstrated stable flux at laboratory scale [51], yet commercial juice processing environments, with their inherent variability in feed composition and frequent cleaning-in-place cycles, can shorten the effective membrane life considerably. Membrane fabrication costs are moderate (PDMS-based pervaporation membranes 20–50 USD m^−2^; nanofiltration membranes 30–150 USD m^−2^, Table 1), but the total cost of ownership is heavily influenced by fouling-related productivity losses. Scale-up challenges, including module design, packing density, process integration, and the discrepancy between laboratory-scale and industrial-scale performance [86].

#### 3.3.2. Sustainability Attributes of Membrane-Based Processes

Beyond their technical performance, membrane separations for essential oil recovery are considered sustainable technologies because they deliver measurable environmental and economic benefits relative to conventional distillation and solvent extraction. Traditional hydrodistillation and steam distillation consume 2–10 kWh per kilogram of oil, mainly to heat and vaporize large quantities of water, and the subsequent condensation step dissipates additional energy [62]. Membrane processes operate at substantially lower temperatures (e.g., 20–40 °C for most pervaporation and nanofiltration operations; Table 1) and principally consume electrical energy for pumping; with negligible thermal energy demand, especially in pervaporation and vapor permeation systems where phase change is localized at the membrane interface rather than in the bulk feed. This low-temperature operation not only cuts the energy footprint but also eliminates the need for organic solvents such as hexane, which are toxic, flammable, and require downstream solvent recovery and disposal. Processes that employ supercritical CO_2_ combined with membranes, as demonstrated for nutmeg essential oil separation, operate entirely without organic solvents and achieve 96.4% retention of the oil components [53], illustrating a closed-loop, solvent-free approach. By retaining the native antioxidant and antimicrobial activity of essential oil components (e.g., >90% retention of limonene and geraniol [48], >95% total antioxidant retention [50]), membrane processes reduce the need for subsequent rectification or additive-based stabilization, further lowering the environmental burden. Economically, the modular nature of membrane units allows incremental capacity expansion and easy integration into existing juice or oil processing lines, avoiding the large capital outlay and footprint of distillation columns. The combination of lower energy consumption, reduced chemical usage, and higher product quality translates into a lower total cost of ownership over the process lifecycle, particularly when the premium market value of high-purity, bioactive essential oils is considered. These attributes align membrane technology with the principles of green chemistry and process intensification, reinforcing its role as a sustainable alternative for the future of essential oil extraction.

### 3.4. The Future and Industrial Development of Membrane Separation Technology

The industrial deployment of membrane technologies for essential oil recovery remains uneven. Among the various processes, organophilic PV using PDMS-based membranes has progressed most rapidly and has already been successfully commercialized for aroma recovery in the fruit juice and beer industries. In contrast, processes such as high-pressure reverse osmosis under supercritical CO_2_ conditions and organic solvent nanofiltration for antioxidant-rich extract purification are still largely confined to pilot-scale studies or niche pharmaceutical applications [52,84]. In contrast, high-pressure reverse osmosis under supercritical CO_2_ (e.g., 96.4% retention of nutmeg oil [53]) and organic solvent nanofiltration of antioxidant extracts (>95% retention of rosemary phenolics [50]) are still limited to pilot or niche pharmaceutical operations. A quantitative comparison with conventional benchmarks demonstrates the competitive advantages of membrane processes: hydrodistillation consumes 2–10 kWh·kg^−1^ of oil and operates at ~100 °C, often causing 30–50% loss of oxygenated terpenes [62,65], whereas PV and NF operate at 20–60 °C with an energy demand of 0.3–1.5 kWh·kg^−1^, derived almost entirely from pumping work. Crucially, membrane systems achieve 80–98% recovery of target volatiles while more than 90% of native antioxidant activity (e.g., limonene and geraniol retention in lemon oil wastewater [48]), without requiring organic solvents. The moderate membrane fabrication cost (20–150 USD·m^−2^, Table 1) and modular design further position them as a sustainable alternative to thermal separation trains.

Despite these advantages, several interrelated challenges impede broad industrial uptake. Membrane fouling and concentration polarization remain the dominant productivity constraints, as exemplified by the flux decline from 15.6 to 7 L·m^−2^·h^−1^ during ultrafiltration of kiwifruit juice [52] and the marked polarization differences between PDMS and PDMS materials [54]. The transition from laboratory-scale systems (3–200 cm^2^) to industrial modules (1–10 m^2^) remains challenging; for example, in beer aroma recovery, scaling from a 28 cm^2^ cell to a conceptual 13 m^2^ system led to flow maldistribution and localized fouling, significantly reducing the overall mass transfer coefficient [84]. Furthermore, real plant extracts contain waxes, polysaccharides, and polyphenol–protein complexes that accelerate fouling in ways not captured by the model solutions prevalent in academic studies. The absence of standardized cleaning procedures specific to essential oil processing, together with uncertainties in membrane replacement frequency, creates economic and operational risks for small and medium-sized producers.

The next generation of membrane processes for essential oil recovery will be shaped by artificial intelligence (AI), digital twins, and adaptive materials. When these computational and materials science advances converge, membrane-based essential oil recovery is expected to evolve into fully integrated, intelligently controlled systems. AI-assisted membrane material design and process optimization [87], and Machine learning approaches for fouling prediction and operational control [88]. Digital twin technologies for process simulation, monitoring, and scale-up [89,90]. Smart membrane systems integrating real-time sensing and adaptive process control [91]. Among the evaluated technologies, organophilic PV using PDMS-based membranes has emerged as the most mature and industrially viable platform. It has been successfully applied for aroma recovery in fruit juice and beer processing, offering high aroma recovery efficiency, reduced solvent consumption, and significantly lower energy requirements compared with conventional distillation processes.

## 4. Applications of Membrane Separation Technology in Plant Essential Oil Processing

Given the wide diversity of plant essential oils and the pronounced differences in molecular weight, polarity, and functional group distribution among their constituent compounds, selective extraction can be achieved through the rational design and selection of membrane materials with tailored physicochemical properties. In this context, membrane separation should not be regarded as a purely physical process, but rather as an integrated system governed by molecular recognition, interfacial selectivity, and mass transfer regulation [92,93,94,95].

The efficiency and quality of essential oil extraction are strongly influenced by both the intrinsic properties of the raw materials and the compatibility of the selected membrane technology. Plant matrices exhibit substantial variability in morphology (e.g., leaves, petals, fruits, and woody tissues) and compositional characteristics, including solid residue content, viscosity, and essential oil concentration [28,64]. Dwi Sapri Ramadhan et al. (2026) conducted research indicating that the plant matrix exhibits significant variability in form and has distinct compositional characteristics that directly affect membrane fouling behavior, mass transfer efficiency, and ultimately influence the process performance, thereby determining the optimal membrane configuration and operating conditions [96].

To provide a systematic framework for process selection, this section categorizes plant raw materials into four major groups: woody and floral plants, herbaceous plants, citrus plants, and spices and aromatic plants [97,98,99,100,101]. For each category, the corresponding membrane separation strategies, process performance, and supporting technologies are critically reviewed. This classification enables a more targeted evaluation of membrane applications and facilitates the rational design of extraction processes for different plant systems.

**Figure 7:**
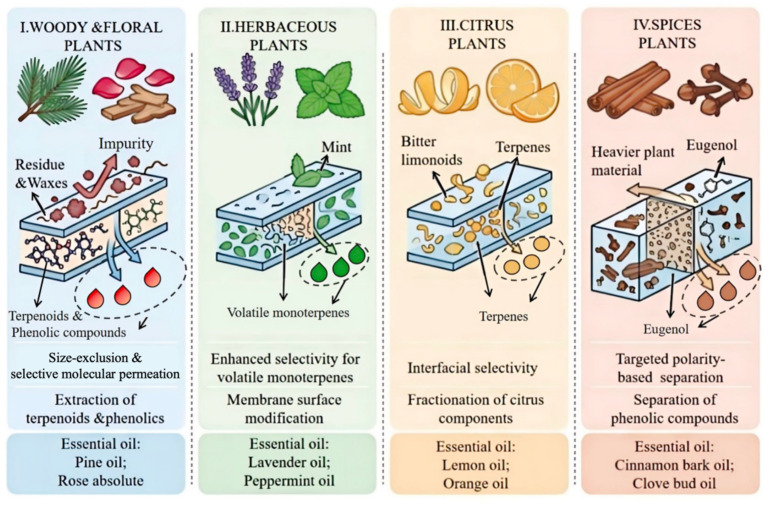
Comparison of Different Membrane Separation Technologies for Four Major Categories of Plants.

A comparative overview of membrane separation technologies applied to these four plant categories is presented in Figure 7, highlighting their respective advantages and application scopes.

### 4.1. Application of Membrane Separation Technology for Extracting Essential Oils from Woody Plants

Woody and floral plants (e.g., rose, jasmine, and osmanthus) are highly valued due to their distinctive aromatic profiles and rich content of bioactive compounds [102,103]. The primary raw materials for essential oil extraction are typically petals and, to a lesser extent, woody tissues. These materials are characterized by low concentrations of volatile components in aqueous extracts, the presence of minor fibrous residues, and a high proportion of thermolabile compounds. Consequently, the key challenge lies in achieving efficient enrichment and purification under mild conditions while preserving sensitive aromatic constituents.

To address these challenges, membrane separation is typically implemented as a multi-stage process integrating pretreatment, clarification, purification, and concentration. Such systems must balance separation selectivity, product recovery, and operational mildness. Pressure-driven membrane processes—including MF, UF, NF, and RO—are commonly employed. Their fundamental principle involves the application of transmembrane pressure to drive solvent transport, while selective separation is achieved via size exclusion and, to some extent, physicochemical interactions between solutes and membrane materials. Stoyanova Y et al. applied nanofiltration to recover polyphenols from Damask rose distillation wastewater. At 10 bar, the DL (Veolia) membrane demonstrated superior fouling resistance and facilitated efficient fractionation, with the retentate exhibited antioxidant activity up to 2.0 times that of the feed [74].

In practical applications, MF and UF are primarily used for clarification and decontamination of crude extracts, effectively removing suspended solids, colloidal impurities, and microorganisms in oil–water emulsions [26]. NF and RO, characterized by molecular weight cut-offs of approximately 100–2000 g·mol^−1^ and <100 g·mol^−1^, respectively, enable further purification and concentration by selectively retaining organic solutes while allowing solvent permeation. This staged separation strategy significantly reduces the energy demand of downstream thermal processes [104].

The extraction of rose essential oil from hydrosol provides a representative example. Initially, coarse filtration is applied to remove large particulates, followed by UF to reduce microbial load and clarify the solution [105]. Subsequent NF enables the removal of small-molecule impurities, such as organic acids, while retaining key aromatic components. Finally, low-temperature reverse osmosis is employed for concentration, thereby enhancing product purity while avoiding thermal degradation associated with conventional distillation. Sabahi et al. [72] conducted a study to evaluate the effects of UF combined with ultraviolet irradiation treatment on the microbial content and chemical composition of rose hydrosol. Gas Chromatography-Mass Spectrometry (GC-MS) analysis indicated that the relative proportions of the main aroma components of the rose water after UF remained basically unchanged, confirming that the UF can serve as a highly reliable clarification and sterilization step, while retaining its characteristic aroma. In addition, NF and UF membranes have been applied to stabilize and concentrate essential oil emulsions, further expanding their functional roles in process integration.

Beyond pressure-driven processes, alternative membrane techniques such as PV and organophilic membranes have also been explored for the selective recovery of volatile aroma compounds [106]. These approaches enable separation under ambient or low-temperature conditions, thereby minimizing the loss of thermolabile constituents and improving overall product quality. Hornyák et al. [49] achieved the selective recovery of aromatic compounds from aqueous solutions using PV membrane technology, demonstrating that this method can effectively minimise the loss of heat-sensitive aroma compounds at low temperatures and improve the quality of aromatic products.

Figure S3 illustrates a typical multi-stage membrane cascade for woody floral essential oil extraction, using rose hydrosol as a model system. The integrated application of MF, UF, NF, and RO not only enhances separation efficiency but also preserves the integrity of sensitive compounds. Overall, this strategy represents a technically feasible and energy-efficient approach for the high-purity recovery of essential oils from woody and floral plant matrices.

Membrane separation technology is widely applied in the processing of rose hydrosol and crude extracts, owing to its ability to effectively enrich bioactive components under mild conditions. Table 2 provides a systematic comparison of membrane separation, steam distillation, and supercritical CO_2_ extraction in terms of operating conditions, extraction efficiency, as well as their respective advantages and limitations. Such comparative analysis offers a scientific basis for the rational selection of processing strategies according to specific product quality requirements and application objectives. Dushkova et al. investigated the application of ultrafiltration membrane technology in the recovery of active components from roses, identifying the key advantages of the membrane separation process: operation at room temperature and low pressure, and high retention of active compounds; He et al. conducted a systematic analysis of the extraction efficiency and technical limitations of steam distillation in the extraction of rose essential oil; Cui et al. optimised the supercritical CO_2_ extraction process for rose essential oil, establishing the extraction yield and suitable applications of this method. The aforementioned studies provide a comprehensive scientific basis for the systematic comparison and targeted selection of these three rose essential oil extraction [73,98,107].

### 4.2. Application of Membrane Separation Technology for Extracting Essential Oils from Herbal Plants

Aromatic herbal plants, including lavender, rosemary, mint, basil, and thyme, produce essential oils with highly complex chemical compositions dominated by volatile terpenes. These include monoterpenes (e.g., linalool and menthol) and oxygenated derivatives, which are primarily responsible for aroma and bioactivity [99]. Krzysztof et al. employed liquid–liquid extraction (LLE), GC and GC–MS to determine the qualitative and quantitative differences in the organic compounds present in narrow-leaved lavender hydrosol. A total of 47 identical compounds were detected in the oil-phase fractions isolated from both the essential oil and the hydrosol. The concentrations of the major components were similar, and the overall composition was highly comparable. Nevertheless, the concentration of aromatic compounds in the hydrosol was significantly lower than that in the essential oil, which limits their direct application [99].

To enhance the utilization of such by-products, enrichment of volatile components is required. Conventional approaches include distillation, solvent extraction, and supercritical CO_2_ extraction; however, these methods often involve high energy consumption or risk of thermal degradation [97,106]. Among them, PV and osmotic evaporation have been widely investigated for the separation and enrichment of volatile compounds from aqueous systems such as hydrolats. Dawiec-Li’sniewska et al. investigated the effectiveness of osmotic vaporisation in concentrating natural aroma compounds from fruit juice hydrolysates. The optimal operating conditions (60 °C, 1.30 dm^3^/min, 0.5 wt% aroma concentration) predict an enrichment factor of 87.56 in the model multi-component system [110]. The results demonstrated that osmotic vaporisation can effectively concentrate volatile aroma compounds, with stable membrane performance and consistent separation efficiency across different operating scales. This confirms that the technology is suitable for the recovery of aroma compounds from dilute aqueous solutions and is feasible for practical application.

In particular, PV operates based on the solution–diffusion mechanism driven by a transmembrane chemical potential difference, enabling selective permeation of volatile organic compounds through hydrophobic membranes. Materials such as PDMS and its modified composites are widely used due to their high hydrophobicity, chemical stability, and selective affinity for nonpolar aroma molecules. Silvestre et al. conducted a systematic review of the application of PV in the separation of essential oil components [64]. The review indicates that, owing to its dissolution–diffusion mechanism and mild operating temperatures(25–40 °C), PV exhibits high selectivity for the separation of volatile aromatic compounds, whilst effectively preserving heat-sensitive components such as linalool. Compared with conventional distillation, PV operates at significantly lower temperatures, thereby minimizing thermal degradation of sensitive compounds.

Overall, PV-based membrane processes demonstrate high selectivity and mild operating conditions for the recovery, dehydration, and fractionation of volatile essential oil components, and offers better separation performance (such as enrichment factor, permeation flux) [106]. This makes them particularly suitable for preserving thermolabile compounds such as linalool, thereby improving both the quality and functional properties of herbal essential oils.

### 4.3. Application of Membrane Separation Technology for Extracting Essential Oils from Citrus Plants

Citrus essential oils (e.g., lemon, orange, and bergamot) are primarily located in the oil glands of fruit peels, especially in the macular area, and are composed mainly of hydrophobic monoterpenes (e.g., limonene) along with oxygenated terpenoids (e.g., linalool and nerol), which contribute significantly to aroma quality [111]. However, crude citrus oils typically require deterpenation to reduce hydrocarbon terpene content and enrich oxygenated compounds, thereby improving fragrance stability and commercial value. Figoli et al. utilized polymer membranes to selectively recover volatile aromatic compounds from bergamot essential oil via vapour permeation, thereby achieving effective deterpenation and the enrichment of oxygen-containing aromatic compounds under mild conditions, demonstrating the potential of membrane technology to enhance the quality and commercial value of citrus essential oils [97].

PV has emerged as one of the most effective membrane technologies for this purpose. The separation mechanism is governed by differences in solubility and diffusivity of components within hydrophobic membranes, allowing selective permeation at ambient or mild temperatures. Common membrane materials include PDMS, polyoctylmethylsiloxane (PDMS), and hybrid membranes incorporating zeolites (e.g., ZIF-8) or ceramic supports, which enhance selectivity and permeability [112]. For example, Yang et al. reported that ZIF-8/PDMS composite membranes exhibited enhanced selectivity toward specific aroma compounds during lemon hydrosol processing, highlighting the sensitivity of membrane systems to molecular structure [48].

In comparison, conventional techniques such as short-path distillation can achieve partial fractionation but often exhibit limited selectivity. For instance, studies on oregano and rosemary essential oils have shown compositional redistribution rather than targeted enrichment of specific compounds [8,113].

Overall, PV offers several advantages over traditional methods, including lower energy consumption, fewer processing steps, and improved preservation of volatile compounds [95]. By exploiting differences in volatility and membrane affinity, PV enables efficient separation of monoterpenes and high-value oxygenated terpenoids, providing a promising route for industrial-scale citrus essential oil refinement.

### 4.4. Application of Membrane Separation Technology for Extracting Essential Oils from Spices and Aromatic Plants

Essential oils derived from spices and aromatic plants (e.g., ginger, garlic, black pepper, cinnamon, and clove) are typically rich in bioactive compounds with relatively high molecular weights, such as phenolics (e.g., eugenol), aldehydes (e.g., cinnamaldehyde), and other functional constituents, often coexisting with pigments and waxy impurities. These systems are commonly processed in organic solvents, posing challenges for conventional separation methods [61,114].

OSN has emerged as an effective approach for such systems, enabling the selective separation of solutes based on molecular size and solvent–membrane interactions. Huang et al. [61] demonstrated that molecularly porous cross-linked membranes (MPCMs) exhibited ultrafast solvent permeance for both polar and nonpolar solvents while maintaining sharp molecular sieving selectivity and excellent long-term stability in aggressive organic media. Shi Xiansong et al. (2022) designed and developed a three-dimensional COF membrane for OSN, which exhibited extremely high stability towards high-concentration feedstock and could operate for approximately 1000 h continuously, with high selectivity and stability [115]. OSN offers several advantages, including solvent resistance, high permeability, and strong selectivity. It is particularly effective for removing macromolecular impurities (e.g., pigments and waxes) while concentrating target compounds such as piperine, cinnamaldehyde, and eugenol.

Common membrane materials include PI, cross-linked PA, PDMS-based composites, and PES. Shi et al. [114] reviewed the latest research progress on OSN membranes, noting that PI, cross-linked PA, PDMS-based composites and PES membranes demonstrate excellent solvent resistance, high permeation rates and outstanding solute-selective separation capabilities in a variety of organic solvent systems, enabling OSN technology to be widely applied in various organic solvent systems. These membranes can operate in both polar and nonpolar organic solvent systems, enabling applications such as terpene removal, decolorization, and fractional concentration.

OSN is an efficient alternative for solvent separation, featuring high solvent permeability and rejection rate. It is particularly suitable for refining processes that require high selectivity and solvent compatibility [114], and is a highly applicable and scalable solution for refining aromatic essential oils.

In summary, membrane separation technology has emerged as a versatile and efficient platform for the extraction and refinement of plant essential oils across diverse botanical sources. By integrating pressure-driven processes (MF, UF, NF, and RO), PV, and OSN, it enables targeted separation based on molecular size, polarity, and volatility. These technologies demonstrate strong adaptability to different plant matrices, including woody floral, herbal, citrus, and spice-derived materials, achieving functions such as clarification, selective enrichment, de-terpenization, and impurity removal. Table 3 summarizes the applicable membrane separation technologies, membrane materials, separation targets and technical advantages for four types of essential oils. Based on existing literature, it clarifies the adaptation rules of their membrane separation technologies, providing a reference for the targeted design of essential oil membrane separation processes. MF and UF serve as pretreatment steps, effectively removing the vast majority of suspended solids and colloidal macromolecules; NF and RO can subsequently concentrate aroma-active oxygenated terpenes with rejections exceeding 80% while partially de-terpenating citrus oils [116]. PV, driven by a transmembrane partial-pressure gradient and operating at 25–40 °C, obtains enrichment factors for key volatile compounds such as linalool and linalyl acetate, reducing specific energy consumption compared with conventional steam distillation. OSN further enables solvent recovery in a single ambient-temperature step. The mild operating conditions, high selectivity, and low energy consumption of membrane processes make them particularly suitable for preserving thermolabile and volatile bioactive compounds. Overall, the synergistic integration of membrane materials and process design provides a robust theoretical and technological foundation for the high-value utilization of plant essential oils.

## 5. Conclusions

This updated systematic review focuses on the theory, classification and performance comparison of membrane separation techniques applied in plant essential oil extraction, and conducts rigorous retrieval and screening from authoritative databases including Web of Science, ScienceDirect, Scopus, PubMed and CNKI, excluding irrelevant literature, duplicate papers, incomplete documents, conference abstracts and obsolete reviews to systematically analyze eligible high-quality research articles. This work clarifies the separation mechanism of membrane technology relying on selective permeability, physical sieving and molecular interaction, compares the application features and separation effects of microfiltration, ultrafiltration, nanofiltration, reverse osmosis and pervaporation membranes, and confirms that membrane separation is a green and efficient method with obvious advantages over traditional purification technologies, which can efficiently remove impurities and realize the high-purity purification and enrichment of plant essential oils. Despite the promising application potential of membrane separation in the high-value and green processing of plant essential oils, this technology still faces several pressing constraints hindering its large-scale industrial promotion, such as severe membrane fouling and concentration polarization, short service life of membrane modules, high manufacturing cost of specialized functional membranes, insufficient adaptability to complex and viscous essential oil extraction systems, and unstable separation efficiency for multi-component essential oils; therefore, future research should focus on the development of novel anti-fouling, high-flux and low-cost membrane materials with modified surface properties, the optimization of integrated separation processes coupled with traditional extraction methods, the improvement of online membrane cleaning and regeneration technologies, the scale-up and continuous operation design of membrane separation equipment, and the exploration of tailored membrane processes for different types of plant essential oils, so as to break through existing technical bottlenecks, reduce production costs and improve process stability, and this review aims to lay a solid theoretical foundation and provide practical guidance for follow-up academic studies, technological innovation and industrialized application in this field. Overall, membrane separation technology demonstrates considerable potential for the sustainable, efficient, and high-quality production of plant essential oils.

## Figures and Tables

**Table 2:** Comparison Table of Different Extraction Methods for Rose Essential Oil.

Extraction Methods	Operating Conditions	Extraction Efficiency	Core Advantages	Main Disadvantages	Applicable Scenarios	Reference
Membrane separation technology	RT LMP	Active ingredient retention: 78–88%	Low-temperature operation Thorough impurity removal Solvent-free High safety	High initial equipment cost Long process flow	Mid-to-high-end rose oil: mass production	[72,73]
Steam distillation	HT AP ET: 2–3 h	Average essential oil yield: 0.035%	Minimal equipment Cost-effective Mature technology	Thermal-sensitive components loss Low yield Long processing time High energy consumption	Low-end rose oil: small-scale production	[98,108]
Supercritical CO_2_ extraction	MT HP Solvent: CO_2_	Max essential oil yield: 8.99%	Higher yield than distillation Solvent-free High active ingredient retention	Initial cost 3–5 times membrane separation High operating cost Limited scalability	High-end rose oil: small-batch production	[107,109]

Notes: RT = room temperature; LMP = medium-low pressure; HT = high temperature; AP = atmospheric pressure; ET = extraction time; MT = medium temperature; HP = high pressure.

**Table 3:** Membrane separation techniques in the extraction of different types of plant essential oils.

Types of Plant Essential Oils	Representative Plants	Adaptive Membrane Technology	Recommended Membrane Materials	Core Separation Target	Key Advantages	References
Woody plants	Rose, jasmine, osmanthus	MF, UF, NF, RO	Conventional polymers PDMS-modified Organophilic	Remove fibers, colloids, microbes, organic acids Retain heat-sensitive actives	Gentle operation High aroma fidelity Anti-fouling Balanced recovery/purity	[26,49,72,104]
Herbaceous plants	Lavender, rosemary, mint	PV	PDMS and modified composite	Enrich volatile terpenoids Concentrate aromatic compounds	Low energy High selectivity Low-temp mitigates thermal degradation	[19,97,99,106]
Citrus plants	Lemon, orange, bergamot	PV	PDMS, PDMS, ZIF-8/PDMS Ceramic-based composite	Remove hydrophobic monoterpenes Enrich oxygenated terpenoids	RT High selectivity Water-compatible Improved flavor stability; structure-sensitive	[48,95,112]
Spices and aromatic plants	Ginger, cinnamon, black pepper	OSN	PI Cross-linked PA PDMS composite PES	Remove pigments, waxes Preconcentrate phenolics, aldehydes	Solvent resistant High permeability High selectivity Polar/non-polar compatible	[61,114]

## Data Availability

No new data were created or analyzed in this study. Data sharing is not applicable to this article.

## Supplementary Materials

The following supporting information can be downloaded at: https://www.mdpi.com/article/10.3390/foods15132283/s1, Figure S1: Regarding the Selection of Raw Materials for Plant Essential Oils, the Various Extraction Methods Used, and the Application of the Final Products; Figure S2: The keyword clustering diagram for extracting plant essential oils using membrane separation technology. (Note: Different colors represent different clusters.); Figure S3: The Membrane Separation Extraction Process for Essential Oils of Woody Flowers (Take Rose Hydrosol as an Example); Table S1: A comparative analysis of nearly 20 academic papers on the membrane separation of plant essential oils.

## Abbreviations

The following abbreviations are used in this manuscript:

HPLC	High-Performance Liquid Chromatography
GC	Gas Chromatography
MF	Microfiltration
UF	Nanofiltration
RO	Reverse Osmosis
PV	Pervaporation
MD	Membrane Distillation
TMP	Transmembrane Pressure
ED	Electrodialysis
RSM	Response Surface Methodology
MMMs	Mixed-Matrix Membranes
MOFs	Metal–Organic Frameworks
PA	Polyamide
PES	Polyethersulfone
PDMS	Polydimethylsiloxane
OSN	Organic Solvent Nanofiltration
PI	Polyimide
AI	Artificial Intelligence
GC-MS	Gas Chromatography-Mass Spectrometry
LLE	Liquid–Liquid Extraction
PDMS	Polyoctylmethylsiloxane
MPCMs	Molecularly Porous Cross-linked Membranes
