# Supplementary material for: Membrane Separation Techniques for Plant Essential Oils: Theory, Performance Comparison, and Application—An Updated Review
Source: Foods. 2026 Jun 25;15(13):2283. doi: 10.3390/foods15132283 (PMC13360812; doi:10.3390/foods15132283)
Supplement: Supplementary file 1 [file foods-15-02283-s001.zip › foods-4334969-supplementary.pdf]

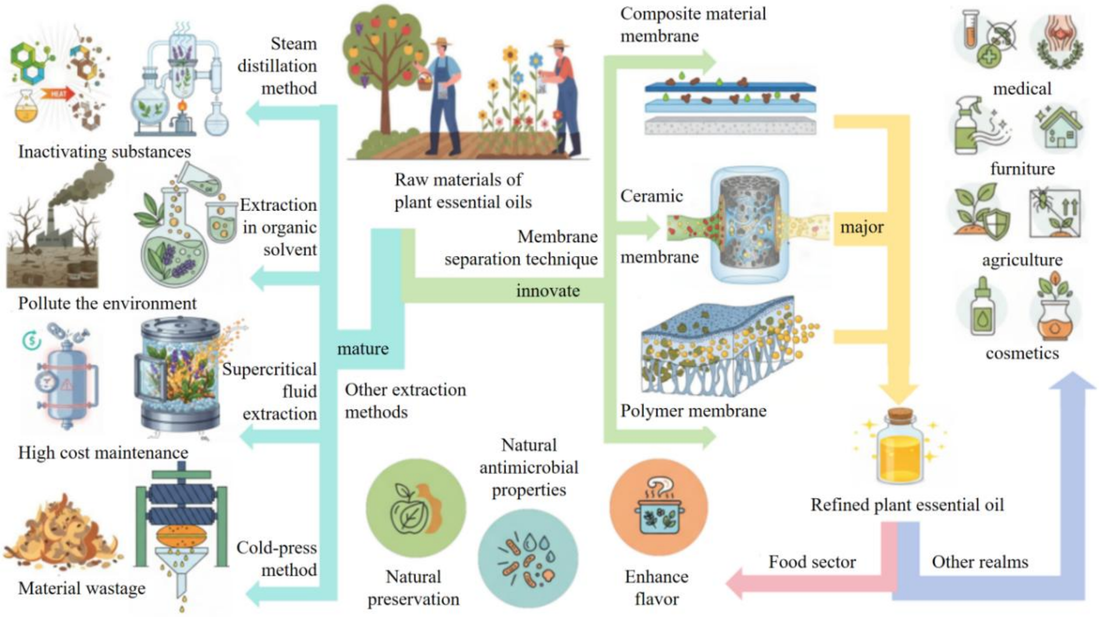


**Figure S1.** Regarding the Selection of Raw Materials for Plant Essential Oils, the Various Extraction Methods Used, and the Application of the Final Products

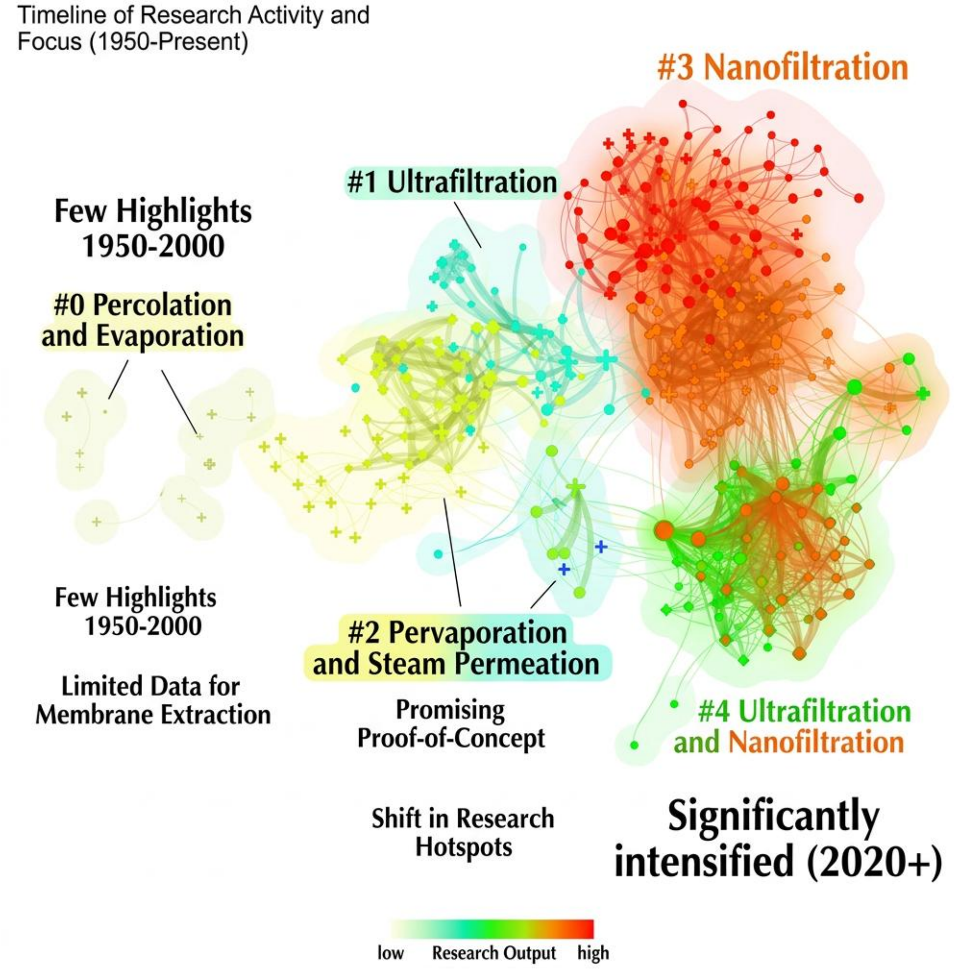


**Figure S2.** The keyword clustering diagram for extracting plant essential oils using membrane separation technology. (Note: Different colors represent different clusters.)

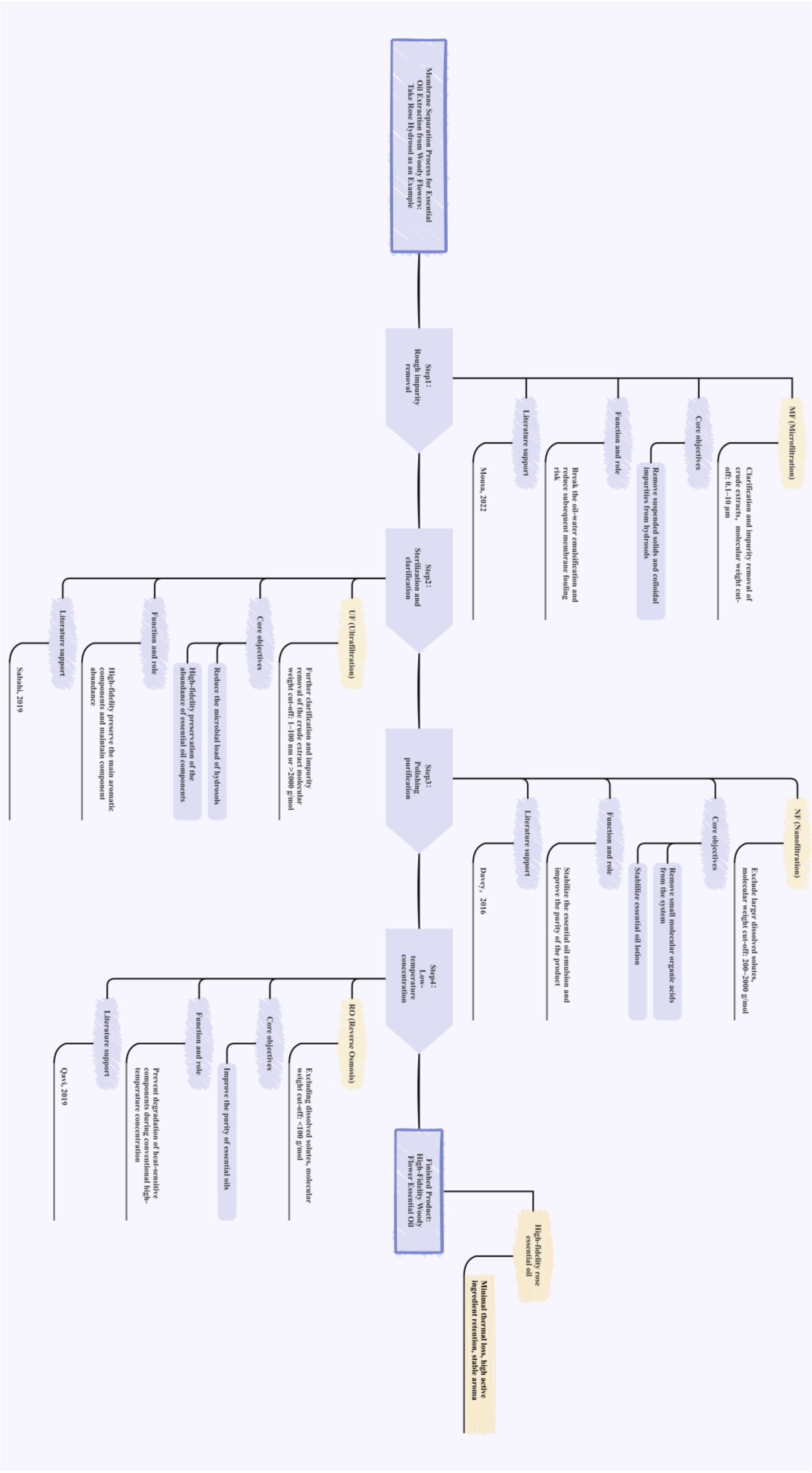


**Figure S3.** The Membrane Separation Extraction Process for Essential Oils of Woody Flowers (Take Rose Hydrosol as an Example)

## **Table S1.** A comparative analysis of nearly 20 academic papers on the membrane separation of plant essential oils

| Literature Title and Authors | Membrane Type | Extracted Plant Essential Oil | Main Chemical Components | Effective Area | Principle | (Total) Membrane Flux | Feed (Optimal) Temperature | Membrane Fabrication Cost (Unit Cost) | Membrane Service Life | Antioxidant Property Retention | Antimicrobial Activity | Antifouling Property | Component Recovery Rate | Total Recovery Rate | Literature Title and Authors | Membrane Type | References |
|---|---|---|---|---|---|---|---|---|---|---|---|---|---|---|---|---|---|
| Pervaporation recovery of aromatic compounds from lemon oil wastewater using a uniform zeolitic imidazolate framework/polydimethylsiloxane composite membrane Dongyi Yang et al. | uniform zeolitic imidazolate framework/polydimethylsiloxane composite membrane | lemon oil (Citrus essential oil) | Linalool, Neral (cis-Citral), Geranial (trans-Citral) | 80 mm² | Pervaporation | 400 – 700 mg • m⁻² • h⁻¹ | 50° C | \$50 – 200/m² | 0-1 year | / | / | / | Linalool: 97.20%, Neral: 96.15%, Geranial: 92.56% (after 9 hours of treatment) | Approx. tens to hundreds of milligrams level | Pervaporation recovery of aromatic compounds from lemon oil wastewater using a uniform zeolitic imidazolate framework/polydimethylsiloxane composite membrane Dongyi Yang et al. | uniform zeolitic imidazolate framework/polydimethylsiloxane composite membrane | [48] |
| Recovery of volatile aroma components from orange juice by pervaporation Abdolreza Aroujalian et al. | PDMS-PVDF-PP composite membrane | Fresh orange juice (Citrus essential oil) | Ethyl Acetate, Ethyl Butyrate, Hexanal, Limonene, Linalool, α-Terpineol | 137.75 cm² | Pervaporation | 100 – 500 g/m² • h | / | \$200 – \$500/m² | 2 – 4 years | / | / | PDMS membrane surface is hydrophobic, has certain antifouling properties against organics, but long-term operation still requires regular cleaning | Ethyl Acetate up to ~18 (varies with temperature and permeate pressure), Ethyl Butyrate approx. 4 – 10, Hexanal approx. 3 – 8, Limonene approx. 2 – 6, Linalool approx. 2 – 5, α-Terpineol approx. 1 – 3 | / | Recovery of volatile aroma components from orange juice by pervaporation Abdolreza Aroujalian et al. | PDMS-PVDF-PP composite membrane | [55] |
| Potentiality of polymeric membranes in aromatherapy: Application to bergamot essential oil A. Figoli et al. | Lab-made membranes: M1: PVDF-HFP, M2: SBS, M3: PSU; Commercial membranes: M4: Cellulose reverse osmosis membrane (Model CD), M5: Polyamide reverse osmosis membrane (Model AD) | Bergamot (Citrus essential oil) | Limonene, Linalool, Linalyl acetate, β-Pinene, α-Pinene, p-Cymene | 20 – 200 cm² | Vapor Permeation (VP) | 0.005 – 0.018 kg/m²h | 30° C | \$100 – \$800/m² | 2-4 years | DPPH radical scavenging rate: 93.19% at concentration 70 mg/mL | / | / | PSU (M3) and Cellulose (M4) membranes showed highest selectivity, completely retaining all oxygenated monoterpenes (e.g., Linalool, Linalyl acetate) | / | Potentiality of polymeric membranes in aromatherapy: Application to bergamot essential oil A. Figoli et al. | Lab-made membranes: M1: PVDF-HFP, M2: SBS, M3: PSU; Commercial membranes: M4: Cellulose reverse osmosis membrane (Model CD), M6: Polyamide reverse osmosis membrane (Model AD) | [97] |
| Selective extraction of natural products with benign solvents and recovery by organophilic pervaporation: fractionation of D-limonene from orange peels — | POMS-PEI organophilic pervaporation membrane (polyoctylmethylsiloxane on polyetherimide) | Orange peel essential oil (mainly D-limonene) | limonene, α-pinene, β-pinene, myrcene, octanal, 3-carene, octanol, nerol, linalool, | 7.065 cm² | Organophilic pervaporation (PV) 20 ° C | PV total flux (mol • m⁻² • h⁻¹): AO 2.960; PPG 3.151; PEG 3.291; Key | 20 ° C | / | / | / | / | PV water flux lower than VD; organophilic membrane has high selectivity, external mass transfer conditions | Enrichment factor β (limonene): AO 11.4; PPG 12.9; PEG 237.8; Other representatives: under PEG conditions myrcene β 38.4, octanal β 24.8, linalool | / | Selective extraction of natural products with benign solvents and recovery by organophilic pervaporation: fractionation of D-limonene from orange peels — | POMS-PEI organophilic pervaporation membrane (polyoctylmethylsiloxane on polyetherimide) | [94] |

| Literature Title and Authors | Membrane Type | Extracted Plant Essential Oil | Main Chemical Components | Effective Area | Principle | (Total) Membrane Flux | Feed (Optimal) Temperature | Membrane Fabrication Cost (Unit Cost) | Membrane Service Life | Antioxidant Property Retention | Antimicrobial Activity | Antifouling Property | Component Recovery Rate | Total Recovery Rate | Literature Title and Authors | Membrane Type | References |
|---|---|---|---|---|---|---|---|---|---|---|---|---|---|---|---|---|---|
| Prashant S. Kulkarni, Carla Brazinha, Carlos A.M. Afonso, João G. Crespo, Green Chemistry (2010) 12, 1990–1994 | | | terpineol | | | component limonene Ji ($mol \cdot m^{-2} \cdot h^{-1}$): AO 0.073; PPG 0.065; PEG 0.051 | | | | | | consistent (stirring 200 rpm) | β 24.9 | | Prashant S. Kulkarni, Carla Brazinha, Carlos A.M. Afonso, João G. Crespo, Green Chemistry (2010) 12, 1990–1995 | | |
| Pervaporation recovery of aromatic compounds from lemon oil wastewater using a uniform zeolitic imidazolate framework/polydimethylsiloxane composite membrane — Shanshan Li, Y. Liu, et al., Separation and Purification Technology, 2023 | ZIF-8/PDMS composite membrane (zeolitic imidazolate framework–polydimethylsiloxane composite membrane) | Aromatic compounds from lemon oil wastewater (Citrus essential oil) | Limonene, β-pinene, γ-terpinene, Geraniol | 28 $cm^2$ | Pervaporation | 560–720 $g \cdot m^{-2} \cdot h^{-1}$ | 40 °C | US $20–50 /m² | > 6 months | Antioxidant components (limonene, geraniol) retention >90 % | Geraniol, limonene retain natural antimicrobial activity | ZIF-8 enhances membrane hydrophobicity and anti-adsorption properties, performance recovery after cleaning ≈ 98 % | Limonene 92 %; β-pinene 85 %; γ-terpinene 88 %; Geraniol 78 % | Overall recovery ≈ 86 % | Pervaporation recovery of aromatic compounds from lemon oil wastewater using a uniform zeolitic imidazolate framework/polydimethylsiloxane composite membrane — Shanshan Li, Y. Liu, et al., Separation and Purification Technology, 2024 | ZIF-9/PDMS composite membrane (zeolitic imidazolate framework–polydimethylsiloxane composite membrane) | [48] |
| Hollow fibre modules for orange juice aroma recovery using pervaporation — Andrew Shepherd, Alberto C. Habert, Cristiano P. Borges, Desalination, 2002 | PDMS (Polydimethylsiloxane) hollow fiber membrane (WSLO and transverse flow modules) | Orange juice aroma (Citrus essential oil) | Ethyl butyrate, Ethanol, water and low concentration aromatic esters | 0.257 $m^2$ | Pervaporation | Aroma 7–8 $g \cdot m^{-2} \cdot h^{-1}$; Water and ethanol 20–30 $g \cdot m^{-2} \cdot h^{-1}$ | 42° C | US $20–50 /m² | >6 months | High (low-temperature operation avoids aromatic oxidation loss) | Not specifically tested; permeate containing citrus aroma has natural antimicrobial properties | Good (WSLO module reduces concentration polarization, stable operating flux) | Ethyl butyrate concentration highest ≈ 50 ppm; Ethanol enrichment factor 3.8; Aroma enrichment factor ≈ 8 | Overall aroma recovery ≈ 80 % | Hollow fibre modules for orange juice aroma recovery using pervaporation — Andrew Shepherd, Alberto C. Habert, Cristiano P. Borges, Desalination, 2003 | PDMS (Polydimethylsiloxane) hollow fiber membrane (WSLO and transverse flow modules) | [51] |
| Application of organic solvent nanofiltration for concentration of antioxidant extracts of rosemary (Rosmarinus officinalis L.) | Duramem™ organic solvent nanofiltration membrane (200, 300, 500 Da) | Rosemary (Rosmarinus officinalis L.) ethanol extract (Spice essential | Rosmarinic acid, Caffeic acid | 56 $cm^2$ | Organic Solvent Nanofiltration (OSN) | 15.4–41.1 $L \cdot m^{-2} \cdot h^{-1}$ (20–40 bar) | 25 ± 1 °C | 80–150 USD/m² | > 6 months | High (concentrate antioxidant capacity decrease < 5 %) | Not involved | Flux decay ≈ 15 % (within two dialysis volumes) | RA ≈ 99.2 %; CA ≈ 95 % | Total antioxidant retention > 95 % | Application of organic solvent nanofiltration for concentration of antioxidant extracts of rosemary (Rosmarinus officinalis L.) | Duramem™ organic solvent nanofiltration membrane (200, 300, 501 Da) | [50] |

| Literature Title and Authors | Membrane Type | Extracted Plant Essential Oil | Main Chemical Components | Effective Area | Principle | (Total) Membrane Flux | Feed (Optimal) Temperature | Membrane Fabrication Cost (Unit Cost) | Membrane Service Life | Antioxidant Property Retention | Antimicrobial Activity | Antifouling Property | Component Recovery Rate | Total Recovery Rate | Literature Title and Authors | Membrane Type | References |
|---|---|---|---|---|---|---|---|---|---|---|---|---|---|---|---|---|---|
| — Pesheva et al., 2011 | | oil) | | | | | | | | | | | | | — Pesheva et al., 2012 | | |
| Separation of nutmeg essential oil and dense $CO_2$ with a cellulose acetate reverse osmosis membrane Cinthia Bittencourt Spricigo et al. | Cellulose Acetate Reverse Osmosis Membrane, Model CF (Osmonics, USA) | Nutmeg essential oil (Spice essential oil) | α-pinene, camphene, limonene, sabinene, β-pinene, myristicin | 3.14 $cm^2$ | Reverse Osmosis (RO) under dense $CO_2$ conditions. | $CO_2$ flux: Linear relationship with ΔP, average permeability 32.087 kg $h^{-1}$ $m^{-2}$ $MPa^{-1}$. | 23, 40, 50 ° C | $30-150/$m^2$ | / | The chemical composition of the essential oil before and after passing through the membrane did not change, meaning components with potential antioxidant activity (e.g., terpenes) were retained. | The cellulose acetate membrane itself does not possess significant inherent antimicrobial activity. | Reversible fouling/concentration polarization observed. • Evidence: $CO_2$ flux decreased with increasing essential oil concentration during separation of essential oil/$CO_2$ mixture (see original Fig.5). • Key finding: After experiment, pure $CO_2$ flux of membrane recovered to initial value (see original Fig.6), indicating fouling is reversible, likely mainly concentration polarization, not irreversible pore clogging or adsorption. • Conclusion: The membrane showed good antifouling recovery under described conditions. | Average retention index as high as 96.4% (standard deviation ± 1.4%) | / | Separation of nutmeg essential oil and dense $CO_2$ with a cellulose acetate reverse osmosis membrane Cinthia Bittencourt Spricigo et al. | Cellulose Acetate Reverse Osmosis Membrane, Model CF (Osmonics, USA) | [53] |
| Pervaporative | PDMS membrane | Beer aroma | Ethyl | Experi | Pervapo | 367– | 20– | 200–300 | 1–2 | High | / | / | Aroma recovery | 18–20% | Pervaporative | PDMS membrane | [84] |

| Literature Title and Authors | Membrane Type | Extracted Plant Essential Oil | Main Chemical Components | Effective Area | Principle | (Total) Membrane Flux | Feed (Optimal) Temperature | Membrane Fabrication Cost (Unit Cost) | Membrane Service Life | Antioxidant Property Retention | Antimicrobial Activity | Antifouling Property | Component Recovery Rate | Total Recovery Rate | Literature Title and Authors | Membrane Type | References |
|---|---|---|---|---|---|---|---|---|---|---|---|---|---|---|---|---|---|
| recovery of aroma compounds in the production of non-alcoholic beers: Incorporation of different condensation strategies into the conceptual design of the process (2024) | pervaporation | compounds | acetate, Isoamyl alcohol, Isobutanol, Propanol, Isoamyl acetate, Acetaldehyde, etc. | ment 28 cm²; Industrial simulation 13 m² | ration | 775 $g\cdot m^{-2}\cdot h^{-1}$ | 40° C | RMB/m² | years | aroma retention; A/E ratio 6.22 consistent with original beer | | | rate 12–21%; Ethyl acetate recovery approx. 47%; Isoamyl alcohol recovery approx. 76% | (overall aromatics) | recovery of aroma compounds in the production of non-alcoholic beers: Incorporation of different condensation strategies into the conceptual design of the process (2025) | pervaporation | |
| Pervaporation-aided Processes for the Selective Separation of Aromas, Fragrances and Essential (AFE) Solutes from Agro-food Products and Wastes — Roberto Castro-Muñoz et al., 2021 | Hydrophobic membranes (PDMS, POMS, PEBA, SBS composite membranes, etc.) | Aromas and essential oil components from apple juice, grape juice, tea extract, tropical fruit juice, bergamot essential oil, pomegranate juice, etc. | Ethyl acetate, Hexanal, Hexenol, Linalool, Limonene, Geraniol, Citronellal, Citronellol, Menthol, etc. (aromatic and terpene compounds) | Laboratory scale (0.01 – 0.1 m²) | Pervaporation | 0.02 – 0.28 $kg\cdot m^{-2}\cdot h^{-1}$ | 20 – 65° C | $3.7 – 112 / m² | / | High (good retention of aromas and antioxidants under low-temperature operation) | Aromatic compounds like vanillin, carvacrol retain their natural antimicrobial activity | Good (PDMS and POMS membranes low fouling tendency, easy cleaning) | Recovery rates for various aromatic components 49 – 100% (depending on system and operation) | Overall recovery approx. 60 – 90% | Pervaporation-aided Processes for the Selective Separation of Aromas, Fragrances and Essential (AFE) Solutes from Agro-food Products and Wastes — Roberto Castro-Muñoz et al., 2022 | Hydrophobic membranes (PDMS, POMS, PEBA, SBS composite membranes, etc.) | [106] |
| Separation of aroma compounds from aqueous solutions by pervaporation using polyoctylmethyl siloxane (POMS) and polydimethyl siloxane (PDMS) membranes — P. Sampranpiboon et al., Journal of Membrane Science, 2000 | POMS (Polyoctylmethyl siloxane) and PDMS (Polydimethylsiloxane) composite membranes | Simulated fruit essential oil components (Ethyl butyrate, Ethyl hexanoate) | Ethyl butyrate ($C_6H_{12}O_2$), Ethyl hexanoate ($C_8H_{16}O_2$), Water | 60 cm² | Pervaporation | ETB: 0.010 – 0.019 $kg\cdot m^{-2}\cdot h^{-1}$; ETH: 0.013 – 0.024 $kg\cdot m^{-2}\cdot h^{-1}$ | 30 ° C | 20 – 50 USD/m² | / | High (low temperature avoids degradation of aromatics) | Not involved | POMS slightly better; PDMS concentration polarization more obvious | POMS: 118 – 281; PDMS: 77 – 234 | ETH recovery highest approx. 85 – 90% | Separation of aroma compounds from aqueous solutions by pervaporation using polyoctylmethyl siloxane (POMS) and polydimethyl siloxane (PDMS) membranes — P. Sampranpiboon et al., Journal of Membrane Science, 2001 | POMS (Polyoctylmethyl siloxane) and PDMS (Polydimethylsiloxane) composite membranes | [54] |
| Integrated membrane process for the production of highly nutritional kiwifruit juice — A. Cassano, A. Figoli, A. Tagarelli, G. Sindona, E. | UF: PVDF tubular membrane (15 kDa, 0.23 m², Koch HFM-251); OD: PP hollow fiber membrane (Liqui-Cel® 1.4 m²); PV: PDMS composite membrane (Pervap 1060) | Kiwifruit juice aroma | methyl butanoate, ethyl butanoate, methyl benzoate, ethyl benzoate, 1-hexanol, 3-hexen-1 | UF 0.23 m²; OD 1.4 m²; PV 183 cm² | Combined membrane process: UF clarification → OD concentration | UF: 15.6 →7 $L\cdot m^{-2}\cdot h^{-1}$; OD: 1.0 →0.47 $kg\cdot m^{-2}\cdot h^{-1}$; PV: $2\times10^{-4}$ – $5\times10^{-4}$ $g\cdot m^{-2}\cdot s^{-1}$ | 25 ° C (UF/OD); PV 20 – 40 ° C | 20 – 50 USD/m² | / | TAA decrease ≈ 12 %; Ascorbic acid decrease ≈ 0.5 % | / | UF slight concentration polarization, recoverable by cleaning >95 %; OD stable. | Esters β ≈ 100; Alcohols β ≈ 10 – 40 | Total aroma retention 90 – 95 % | Integrated membrane process for the production of highly nutritional kiwifruit juice — A. Cassano, A. Figoli, A. Tagarelli, G. Sindona, E. | UF: PVDF tubular membrane (15 kDa, 0.23 m², Koch HFM-251); OD: PP hollow fiber membrane (Liqui-Cel® 1.4 m²); PV: PDMS composite membrane (Pervap 1061) | [52] |

| Literature Title and Authors | Membrane Type | Extracted Plant Essential Oil | Main Chemical Components | Effective Area | Principle | (Total) Membrane Flux | Feed (Optimal) Temperature | Membrane Fabrication Cost (Unit Cost) | Membrane Service Life | Antioxidant Property Retention | Antimicrobial Activity | Antifouling Property | Component Recovery Rate | Total Recovery Rate | Literature Title and Authors | Membrane Type | References |
|---|---|---|---|---|---|---|---|---|---|---|---|---|---|---|---|---|---|
| Drioli, Desalination, 189 (2006) 21 – 30 | | | -ol, (E)-2-hexen-1-ol | | → PV aroma recovery. | | | | | | | | | | Drioli, Desalination, 189 (2006) 21 – 31 | | |
| Recovery of aroma compounds from model solution by pervaporation membrane — L. Hornyák, Á.N. Hornyák-Holczman, E. Márki, G. Vatai, Periodica Polytechnica Chem. Eng., 58(1) (2014) 15 – 19 | PERVAP 1060 commercial organophilic PDMS flat-sheet membrane (Sulzer), effective area 131 cm² | Apple aroma model solution (ethanol, ethyl acetate, n-butanol, i-butanol, i-amyl alcohol) | ethanol; ethyl acetate; n-butanol; i-butanol; i-amyl alcohol | 131 cm² | Pervaporation | 0.10 – 0.50 kg·m⁻²·h⁻¹ (20 – 60 ° C) | 20 / 40 / 60 ° C | 20 – 50 USD·m⁻² | > 6 months | / | / | Liquid phase resistance minimal, membrane resistance dominant; feed flow rate and initial concentration influence not significant. | ethanol 15.9 – 96.1; i-butanol 4.1 – 64.7; i-amyl alcohol 0.9 – 24.1; n-butanol 0.5 – 12.6; ethyl acetate 1.2 – 7.0 | Aroma recovery rate 90 – 95 % (highest at 60 ° C) | Recovery of aroma compounds from model solution by pervaporation membrane — L. Hornyák, Á.N. Hornyák-Holczman, E. Márki, G. Vatai, Periodica Polytechnica Chem. Eng., 58(1) (2014) 15 – 20 | PERVAP 1060 commercial organophilic PDMS flat-sheet membrane (Sulzer), effective area 131 cm131 | [107] |
| Aroma compounds recovery of tropical fruit juice by pervaporation: membrane material selection and process evaluation — C.C. Pereira, J.R.M. Rufino, A.C. Habert, R. Nobrega, L.M.C. Cabral, C.P. Borges, Journal of Food Engineering, 66 (2005) 77 – 87 | Pervap 1060 (PDMS), Pervap 1070 (filled PDMS), Lab-made EPDM flat-sheet/hollow fiber membranes, Lab-made EVA membrane | Tropical juice aroma (pineapple juice aroma model system) | Ethyl acetate (EA), Ethyl butanoate (EB), Ethyl hexanoate (EH), 1-octen-3-ol (OCT) | Flat-sheet: 4.5×10⁻³ m²; HF EPDM: 6.22×10⁻⁴ m²; Scale-up simulation: 80 – 1000 m² | Pervaporation | Water flux: Pervap 1060 = 94.4(88.2)×10⁻³ kg·h⁻¹·m⁻²; Pervap 1070 = 37.3(31.6)×10⁻³; EPDM = 12.7(5.6)×10⁻³; EVA = 15.4(12.1)×10⁻³ (25 ℃) | 25 ℃ | 20 – 60 USD·m⁻² | / | / | / | EPDM / EVA low water flux, low concentration polarization; membrane resistance dominant. | Pervap 1070: EA 124.4, EB 410, EH 213.2; EPDM HF: EA 125.3, EB 516, EH 1161; PDMS HF: EA 96.8, EB 516, EH 194 | Engineering simulation: Recovery rate 0.66 – 0.90, corresponding required membrane area 80 – 1000 m² | Aroma compounds recovery of tropical fruit juice by pervaporation: membrane material selection and process evaluation — C.C. Pereira, J.R.M. Rufino, A.C. Habert, R. Nobrega, L.M.C. Cabral, C.P. Borges, Journal of Food Engineering, 66 (2005) 77 – 88 | Pervap 1060 (PDMS), Pervap 1071 (filled PDMS), Lab-made EPDM flat-sheet/hollow fiber membranes, Lab-made EVA membrane | [57] |
| Pervaporative recovery of volatile aroma compounds from fruit juices — C.C. Pereira, C.P. Ribeiro Jr., R. Nobrega, C.P. Borges, Journal of Membrane Science, 274 (2006) 1 – 23 | Review of organophilic membranes: PDMS (Pervap 1060/1070), POMS, PEBA, EPDM, EVA, Silicalite-filled PDMS, POMS-PEI/PVDF, etc. (mainly PDMS) | Fruit juice aromas (volatile esters, alcohols, aldehydes from apple, pineapple, passion fruit, orange, strawberry model solutions and | Frequent components: ethyl acetate, ethyl butanoate, ethyl hexanoate, 1-octen-3-ol, linalool, hexanal, benzaldehyde, S-methyl thiobutan | Lab flat-sheet typical 0.004 – 0.02 m²; Hollow fiber 6×10⁻⁴ – 1×10⁻² m²; Engineering simula | Pervaporation | PDMS type: Water 50 – 120 g·m⁻²·h⁻¹; Ester aromatics during enrichment 1×10⁻⁴ – 2×10⁻⁴ g·m⁻²·s⁻¹; Filled/ | 25 – 40 ° C | / | >6 months | / | / | Feed side liquid film resistance often accounts for 20 – 80 %; high Re (5,000 – 22,000) can compress liquid film; PDMS with high water content requires larger condensatio | Typical ester enrichment β: PDMS-1060/1070 100 – 1000; POMS 100 – 1200; EPDM/EVA for highly hydrophobic esters β can reach 500 – 1100; Silicalite-filled PDMS significantly reduces water permeance. | Industrial condensate/juice aroma overall recovery generally 80 – 95 %, requires multi-stage condensation or increased membrane area to approach 95 %. | Pervaporative recovery of volatile aroma compounds from fruit juices — C.C. Pereira, C.P. Ribeiro Jr., R. Nobrega, C.P. Borges, Journal of Membrane Science, 274 (2006) 1 – 24 | Review of organophilic membranes: PDMS (Pervap 1060/1071), POMS, PEBA, EPDM, EVA, Silicalite-filled PDMS, POMS-PEI/PVDF, etc. (mainly PDMS) | [89] |

| Literature Title and Authors | Membrane Type | Extracted Plant Essential Oil | Main Chemical Components | Effective Area | Principle | (Total) Membrane Flux | Feed (Optimal) Temperature | Membrane Fabrication Cost (Unit Cost) | Membrane Service Life | Antioxidant Property Retention | Antimicrobial Activity | Antifouling Property | Component Recovery Rate | Total Recovery Rate | Literature Title and Authors | Membrane Type | References |
|---|---|---|---|---|---|---|---|---|---|---|---|---|---|---|---|---|---|
| | | industrial condensates) | oate, etc. (69 aromas summarized in Table 2) | tion requires 80–1000 $m^2$ (for feed 3000 $kg \cdot h^{-1}$) | | more hydrophobic membranes water flux 5–40 $g \cdot m^{-2} \cdot h^{-1}$; PEBA/EPDM flux lower but selectivity higher. | | | | | | n load. | | | | | |
| Nanofiltration-Assisted Concentration Processes of Phenolic Fractions and Carotenoids from Natural Food Matrices — Roberto Castro-Muñoz, Separations 11 (2024) 64 | NF commercial membranes: NF270, NF90, ETNA01PP, Desal DK/DL, NP010/NP030, Nadir N30F, HydraCoRe 70 pHT, Duramem 200/300/500, Starmem 240, etc. (polyamide, polysulfone, fluoropolymer) | Phenolics and carotenoids from plant sources (wine pomace, olive wastewater, citrus press liquor, pomegranate peel, berries, chicory/artichoke, South American tropical fruit byproducts, etc.) | Phenolics: quercetin, catechin, epicatechin, chlorogenic acid, caffeic acid, ellagic acid, anthocyanins; Carotenoids: β-carotene, lycopene, lutein, zeaxanthin (varies by source) | Laboratory: 0.004–0.02 $m^2$; Hollow fiber/spiral wound: $6\times10^{-4}$–$1\times10^{-2}$ $m^2$; Industrial simulation: 80–1000 $m^2$ (feed 3000 $kg \cdot h^{-1}$) | Pressurized nanofiltration fractionation | Flux mostly 5–40 $L \cdot m^{-2} \cdot h^{-1}$ (high concentration byproducts); Water flux can reach 50–120 $g \cdot m^{-2} \cdot h^{-1}$; Some lab-scale NF270 at 3 MPa can reach 20–50 $L \cdot m^{-2} \cdot h^{-1}$. | 20–45 °C | 30–80 $USD \cdot m^{-2}$ | > 6 months | / | / | Fouling/concentration polarization accounts for 20–80 % mass transfer resistance; increasing cross-flow velocity, pH control, sequential membrane train can significantly reduce; most cases recoverable by cleaning. | Rejection for various source phenolics/anthocyanins 88–100 %; Wine pomace up to 100 %; Citrus press liquor 70–90 %; Pomegranate/berries 90–99 %; Carotenoid NF/OSN concentration 90–99 %. | Overall recovery generally 80–98 %, integrated processes (MF/UF+NF) and multi-stage condensation/resin can →≈ 100 % (wine pomace/olive wastewater cases). | Nanofiltration-Assisted Concentration Processes of Phenolic Fractions and Carotenoids from Natural Food Matrices — Roberto Castro-Muñoz, Separations 11 (2024) 65 | NF commercial membranes: NF270, NF90, ETNA01PP, Desal DK/DL, NP010/NP030, Nadir N30F, HydraCoRe 70 pHT, Duramem 200/300/500, Starmem 241, etc. (polyamide, polysulfone, fluoropolymer) | [56] |
| Separation of nutmeg essential oil and dense $CO_2$ with a cellulose acetate reverse osmosis membrane — C.B. Spricigo, A. Bolzan, R.A.F. Machado, L.H.C. Carlson, J.C.C. Petrus, Journal of Membrane Science, 188 (2001) 173–179 | Cellulose Acetate (CF, Osmonics) Reverse Osmosis (RO) membrane | Nutmeg essential oil / Dense $CO_2$ mixture | α-pinene, camphene, limonene, sabinene, β-pinene, myristicin | 3.14 $cm^2$ (dead-end cell) | 12 MPa upstream pressure; transmembrane pressure difference 1–4 MPa, $CO_2$ permeates, essential oil | $CO_2$ permeability 32.087 ±2.767 $kg \cdot h^{-1} \cdot m^{-2} \cdot MPa^{-1}$ (flux linear with ΔP) | 23 / 40 / 50 °C (temperature effect not significant; high ΔP increases flux) | 20–60 $USD \cdot m^{-2}$ | ⩾ 180 h | / | / | Reversible fouling/polarization: $CO_2$ flux decreases when essential oil present, recovers later. | Not separated by component; GC–MS composition unchanged before and after. | Average retention 96.4% (±1.4%), insensitive to temperature/concentration/ΔP. | Separation of nutmeg essential oil and dense $CO_2$ with a cellulose acetate reverse osmosis membrane — C.B. Spricigo, A. Bolzan, R.A.F. Machado, L.H.C. Carlson, J.C.C. Petrus, Journal of Membrane Science, 188 (2001) 173–180 | Cellulose Acetate (CF, Osmonics) Reverse Osmosis (RO) membrane | [53] |

| Literature Title and Authors | Membrane Type | Extracted Plant Essential Oil | Main Chemical Components | Effective Area | Principle | (Total) Membrane Flux | Feed (Optimal) Temperature | Membrane Fabrication Cost (Unit Cost) | Membrane Service Life | Antioxidant Property Retention | Antimicrobial Activity | Antifouling Property | Component Recovery Rate | Total Recovery Rate | Literature Title and Authors | Membrane Type | References |
|---|---|---|---|---|---|---|---|---|---|---|---|---|---|---|---|---|---|
| | | | | | retained | | | | | | | | | | | | |
| Structure regulation of PDMS coating on PTFE membrane surface to achieve efficient separation of gaseous peppermint aromatic water — Qin Liu, Xin Liu, Bo Wu*, Changge Wang, Tiantian Li, Wancheng Li, Yun Huang, Yao Li, Haiyang Yan, Chuanrun Li**, Applied Surface Science 665 (2024) 160354 | PDMS/PTFE composite membrane (PTFE base membrane with structurally regulated dense PDMS coating; vapor permeation membrane) | Peppermint aromatic water (vapor phase) | L-menthol; L-menthone; menthyl acetate (permeate composition 49.16%, 26.3%, 5.46% respectively) | 21.19 $cm^2$ | Vapor permeation | Essential oil flux: 70.8–155.6 $g \cdot m^{-2} \cdot h^{-1}$ (90–105 ℃, first 3 h); 76.4–140.1 (4–5 h); 74.3–129.2 (6 h); Table 3 gives representative value 134.5 $g \cdot m^{-2} \cdot h^{-1}$; Water nearly 0 (first 6 h). | 105 ℃ | / | / | / | / | First 6 h almost no water permeation; >12 h wetting/clogging causing separation factor decrease; Contact angle: Water 139°, Oil 27° (optimal conditions). | Main pharmacologically active components reached European Pharmacopoeia range; Maximum separation factor 668; Permeability: L-menthol>P_menthone>menthyl acetate≫water. | Essential oil recovery 17.1% within 6 h (105 ℃, 65 kPa), overall recovery depends on run time and condensation (example for 6 h operation). | Structure regulation of PDMS coating on PTFE membrane surface to achieve efficient separation of gaseous peppermint aromatic water — Qin Liu, Xin Liu, Bo Wu*, Changge Wang, Tiantian Li, Wancheng Li, Yun Huang, Yao Li, Haiyang Yan, Chuanrun Li**, Applied Surface Science 665 (2024) 160355 | PDMS/PTFE composite membrane (PTFE base membrane with structurally regulated dense PDMS coating; vapor permeation membrane) | [85] |

This table summarizes various membrane separation techniques, membrane materials, separation targets, product contents, membrane parameters, membrane performance, and technical advantages applicable to various essential oils. Based on the existing literature, it clarifies the adaptation rules of the membrane separation techniques, providing a reference for the classification of essential oil membrane separation processes.
